# Methionine Synthase Positively Regulates Plant Defence to Both RNA and DNA Viruses and Is Useful for Developing Broad‐Spectrum Antiviral Resistance in Crops

**DOI:** 10.1111/pbi.70618

**Published:** 2026-02-23

**Authors:** Zhaohui Wang, Kunpu Zhang, Chi Zhang, Jin Yang, Bei Li, Lina Wang, Zhenghao Shi, Rui Guo, Shuai Zhang, Kaiqi Gao, Jianing Li, Xiaohuan Jin, Xiang Ji, Huihui Bi, Liyuan You, Huaibing Jin, Daowen Wang

**Affiliations:** ^1^ State Key Laboratory of High‐Efficiency Production of Wheat‐Maize Double Cropping, Henan Center for Crop Genomics and Rice Engineering, College of Agronomy Henan Agricultural University Zhengzhou China; ^2^ National Wheat Engineering Research Center, College of Agronomy Henan Agricultural University Zhengzhou China; ^3^ The Shennong Laboratory Zhengzhou China; ^4^ State Key Laboratory for Biology of Plant Diseases and Insect Pests, Institute of Plant Protection Chinese Academy of Agricultural Sciences Beijing China

**Keywords:** antiviral defence, broad spectrum resistance, methionine synthase, plant viruses, tobacco, viral suppressor of gene silencing, wheat

## Abstract

Plant viruses frequently cause severe economic losses in worldwide crop production. Developing broad‐spectrum resistance is the most efficient approach for controlling plant viral diseases. In this work, we found that the 17K protein of barley yellow dwarf viruses (BYDVs), which has multiple functions in viral pathogenesis including acting as a viral suppressor of gene silencing (VSR), interacted with plant methionine synthase (MS), the last enzyme in the methionine cycle. Silencing *HvMS* gene expression enhanced BYDV symptoms and viral gene expression in barley. In contrast, overexpressing *HvMS1* in wheat, another important host of BYDVs, attenuated disease symptoms and decreased viral genome proliferation. Interestingly, the γb VSR of barley stripe mosaic virus (BSMV) also interacted with HvMS protein, and *HvMS1* overexpression lines likewise exhibited improved BSMV resistance. Further investigations uncovered that the VSRs of potato virus X (PVX) and tobacco rattle virus (TRV) could interact with the MS protein of *Nicotiana benthamiana*; lowering *NbMS* gene expression by genome editing reduced tobacco resistance to PVX and TRV, whereas the reverse was observed in *HvMS1* overexpression tobacco lines. Finally, we showed that HvMS1 could counteract the VSR function of 10 distinct RNA and DNA viruses by obstructing their ability to revive GFP expression in 16c tobacco, suggesting that plant MS protein may act broadly in disrupting the anti‐gene silencing activities of VSRs. Altogether, our data suggest that plant MS protein positively regulates host defence to diverse viruses through inhibiting their VSRs, thus providing a promising target for engineering broad‐spectrum antiviral resistance in crops.

## 1 Introduction

With over 2000 species characterised and causing an estimated $30 million crop losses per annum in the world, plant viruses constitute a group of highly damaging phytopathogens (Jones and Naidu 2019; Savary et al. 2019; Tatineni and Hein 2023). Deployment of resistant cultivars is the most economical and environmentally friendly means for controlling plant viral diseases. But the development of such cultivars requires a sound understanding of the resistance genes active in different virus‐host combinations (Savary et al. 2019; Tatineni and Hein 2023). This can be time consuming and costly, considering that crops are mostly attacked by multiple RNA and DNA viruses and many different viral strains that frequently vary in pathogenicity (Jones and Naidu 2019; Savary et al. 2019; Tatineni and Hein 2023). Consequently, it is highly desirable to identify naturally evolved broad‐spectrum resistance (BSR) genes that are effective in different crops against multiple viruses and their strains (Li et al. 2020; Tatineni and Hein 2023). However, the knowledge of such antiviral BSR genes is still very limited.

To date, most studies on natural antiviral BSR genes are concerned with the resistance against different strains of a single virus or a few RNA and/or DNA viruses. For example, the *JAX1* gene, encoding a jacalin‐type lectin, confers resistance to different potexviruses and their strains by interacting with and inhibiting the viral replicase protein (Okano et al. 2020; Yoshida et al. 2019). The Sw‐5b innate immune receptor conveys BSR to American‐type tospoviruses by binding to a conserved 21‐amino acid peptide in the viral movement protein NSm, thus resulting potent effector‐triggered immunity (ETI) (Zhu et al. 2017). A large body of investigations have shown that natural mutations of protein translation elongation factors eIF4E or eIF4G lead to BSR against potyviruses, bymoviruses, carmoviruses, or fijiviruses, as these host proteins are co‐opted by the viruses for translating their proteins (Machado et al. 2017; Sanfaçon 2015; Zhou et al. 2024). OsAGO18 bestows BSR to two distinct RNA viruses by acting as a miRNA sequester to enhance OsAGO1‐controlled antiviral defence in rice (Wu et al. 2015). The rice *Flotillin1* gene is found required for cell‐to‐cell movement of three discrete RNA viruses and knocking out *Flotillin1* results in BSR against the three viruses by impeding their spread in host plants (Ge et al. 2023). The DEAD‐box RNA helicase RH20 positively regulates RNA interference (RNAi)‐based defence of *Nicotiana benthamiana* to divergent RNA viruses by promoting the amplification of virus derived small‐interfering RNAs (vsiRNAs) (Wen et al. 2024). As for plant DNA viruses, the NIK1 receptor‐like kinase has been found to confer antiviral defence against bipartite begomoviruses by activating pattern‐initiated immunity (PTI) (Teixeira et al. 2019; Zorzatto et al. 2015). A pokeweed antiviral protein originated from *Phytolacca americana* downregulates the spread of cauliflower mosaic virus (a DNA virus) as well as two divergent RNA viruses, i.e., potato virus X (PVX) and cucumber mosaic virus (CMV), by depurination of ribosomal RNA and viral RNA, thus rendering translation suppression of viral genes (Aitbakieva and Domashevskiy 2016; Domashevskiy and Cheng 2018). A recent study reports a general strategy for developing antiviral BSR using a single engineered nucleotide‐binding leucine‐rich‐repeat (NLR) protein, which may be deployed to breed crops resistant to diverse RNA and DNA viruses (Wang et al. 2025). Although still limited in number, the above studies demonstrate that it is possible to mine BSR genes against multiple plant viruses and their strains. A greater understanding of the molecular processes operating during virus‐host interactions, e.g., expression, replication, and cell‐to‐cell movement of viral genomes, host antiviral RNAi defence, activation of PTI, and function of NLR‐mediated ETI, may increase the chance of identifying such genes.

To aid their infections, plant viruses generally encode one or more suppressors of host gene silencing machinery that orchestrates potent RNAi‐based defence against viral gene expression and genome replication; these suppressors, commonly referred as VSRs, are often multifunctional and important determinants of viral pathogenicity (Burgyán and Havelda 2011; Guo et al. 2019; Voinnet et al. 1999; Zhao and Guo 2022). For instance, the 17K protein of barley yellow dwarf viruses (BYDVs), a group of closely related luteoviruses with single‐stranded RNA genomes and infecting barley, wheat, oat, and many other cereal grasses, functions as a VSR, a movement protein (MP), and an inhibitor of host mitosis (Chay et al. 1996; Fusaro et al. 2017; Jin et al. 2020, 2022; Shakir et al. 2024; Tian et al. 2024). To survive virus attack, plants have evolved many ways to counteract the functions of VSRs (Li and Ding 2006; Zhao and Guo 2022). The first one is to develop innate immune receptors capable of recognising VSRs, with their interaction provoking ETI (Zhao et al. 2016; Sett et al. 2022; Wu et al. 2022). The second one is to degrade VSRs through the ubiquitin‐proteasome system or the autophagy pathway (Chen et al. 2024; Hafrén et al. 2018; Jiang et al. 2021; Li et al. 2022; Sharma et al. 2025; Shukla et al. 2022; Tong et al. 2023; Yang et al. 2025). The third one is to modify VSRs post‐translationally, with the modified VSRs functionally altered or even becoming an enhancer of host antiviral RNAi (Jin et al. 2022; Yue et al. 2022). The last one is to evolve specific inhibitors that capable of directly binding and inactivating VSRs (Chen et al. 2017; Nakahara et al. 2012; Xu et al. 2022). One well‐studied example of such inhibitors is the tobacco calmodulin‐like protein rgs‐CaM that interacts with potyviral HC‐Pro and cucumoviral 2b VSRs and promote their degradation through autophagy (Jeon et al. 2017; Nakahara et al. 2012). Similarly, the maize violaxanthin deepoxidase ZmVDE and triacylglycerol lipase ZmTGL interact with and thereby disrupt the HC‐Pro VSR of sugarcane mosaic virus (Chen et al. 2017; Xu et al. 2022). These VSR inhibitors are of great scientific and applied importance because they can aid deeper analysis of the fundamental principles underlying host antiviral RNAi defence and represent potential BSR genes for improving crop resistance against multiple viruses. Hence, further efforts are needed to study their occurrence and functional mechanisms in more virus‐host combinations.

Owing to their extensive damages to global cereal crop productions, BYDVs and the yellow dwarf diseases caused by them have received substantial attention from worldwide researchers (Miller and Lozier 2022). Breeding resistant varieties is a primary task of international BYDV research, whose success depends on the availability of more and diverse resistance genes (Aradottir and Crespo‐Herrera 2021). A number of genetic loci have been identified and used for developing BYDV resistant barley and wheat crops (Bian et al. 2025; Choudhury et al. 2019; Pidon et al. 2024; Zhang et al. 2009). Engineered resistance, developed based on understanding the molecular pathogenesis of viruses, are also promising for fortifying BYDV resistance through biotechnological breeding (Bonning et al. 2014; Jin et al. 2022; Wang et al. 2000, 2024). In previous studies, we found that the 17K protein of BYDVs, being a conserved VSR among luteoviruses and a key determinant of viral pathogenesis, is useful for mining the genes and processes beneficial for enhancing host antiviral resistance (Jin et al. 2020, 2022; Wang et al. 2024). In this work, we discovered that BYDV 17K interacted with barley and wheat methionine synthase (MS) enzymes. In higher plants, MS enzymes, conserved in both monocot and dicot species, are often encoded by multigene families. They regulate plant growth, development, and responses to environmental factors through catalysing the final step of methionine cycle (MTC) and thereby affecting the biosynthesis of many important metabolites, e.g., methionine (MET), S‐adenosyl methionine (SAM), and ethylene (Mäkinen and De 2019; Gonzalez and Vera 2019; García‐Valencia et al. 2025; Ju et al. 2020; Pattyn et al. 2021; Ravanel et al. 2004; Yan et al. 2019; Zhai et al. 2022). Our genetic and molecular analysis indicated that MS protein positively regulates barley and wheat defence against BYDV infection. Importantly, we demonstrate that transgenic overexpression of *HvMS1* led to elevated resistance to diverse RNA and DNA viruses in wheat and *N. benthamiana* plants, suggesting that MS protein likely represents a valuable target for developing antiviral BSR in different crops. Furthermore, we showed that MS could interact with the VSRs of both plant RNA and DNA viruses and suppressed their anti‐gene silencing activities. This provides a novel clue for further studying and improving the molecular basis of MS‐mediated broad spectrum antiviral resistance in crop plants.

## 2 Results

### 2.1 BYDV 17K VSR Interacts With *Arabidopsis*, Barley and Wheat MS Protein

To identify potential interacting proteins of BYDV 17K VSR, we performed immunoprecipitation‐coupled mass spectrometry (IP‐MS) analysis using the transgenic *Arabidopsis* line tA_17K‐GFP (Jin et al. 2020; Wang et al. 2024). Among the peptides identified, six were uniquely mapped to 5‐methyltetrahydropteroyltriglutamate‐homocysteine methyltransferase 1 (AtMS1, encoded by the locus *AT5G17920*, Figure S1a). Subsequently, we verified the interaction of 17K with AtMS1 using yeast two hybrid (Y2H) and split luciferase complementation (SLC) assays (Figure S1b,c). Co‐immunoprecipitation (Co‐IP) assays, conducted using tA_17K‐GFP and the control transgenic line (tA_GFP) expressing free GFP, further validated such interaction (Figure S1d). Searching *Arabidopsis* genome revealed that *AtMS1* has two homologues, *AtMS2* and *AtMS3* (Table S1). Furthermore, there exist varying numbers of *MS1* homologues in barley ( *Hordeum vulgare* , *HvMS1* and *HvMS2*), hexaploid common wheat ( *Triticum aestivum* , *TaMS1A*, *TaMS1B* and *TaMS1D*; *TaMS2A*, *TaMS2B* and *TaMS2D*), *Zea mays* (*ZmMS1*, *ZmMS2* and *ZmMS3*), *Oryza sativa* (*OsMS1* and *OsMS2*), and *N. benthamiana* (*NbMSa—NbMSh*) (Table S1). The deduced MS proteins were similar to each other (identity > 48%, similarity > 62%), with their predicted molecular mass mainly around 84 kDa (Table S1). Consistently, a polyclonal antibody, prepared in this work using the bacterially expressed HvMS1 protein, could recognise endogenous MS proteins in barley, common wheat, and *N. benthamiana* (Figure S1e,f). Furthermore, phylogenetic analysis using the deduced amino acid sequences showed two clusters of MS proteins from monocot and dicot plants, respectively (Figure S1g). The expression and function of multiple *MS* members have been shown in the model plant *Arabidopsis* (Gonzalez and Vera 2019; Ju et al. 2020; Yan et al. 2019). But much less functional studies have been reported on the *MS* genes in the crop plants such as barley and wheat.

**FIGURE 1:**
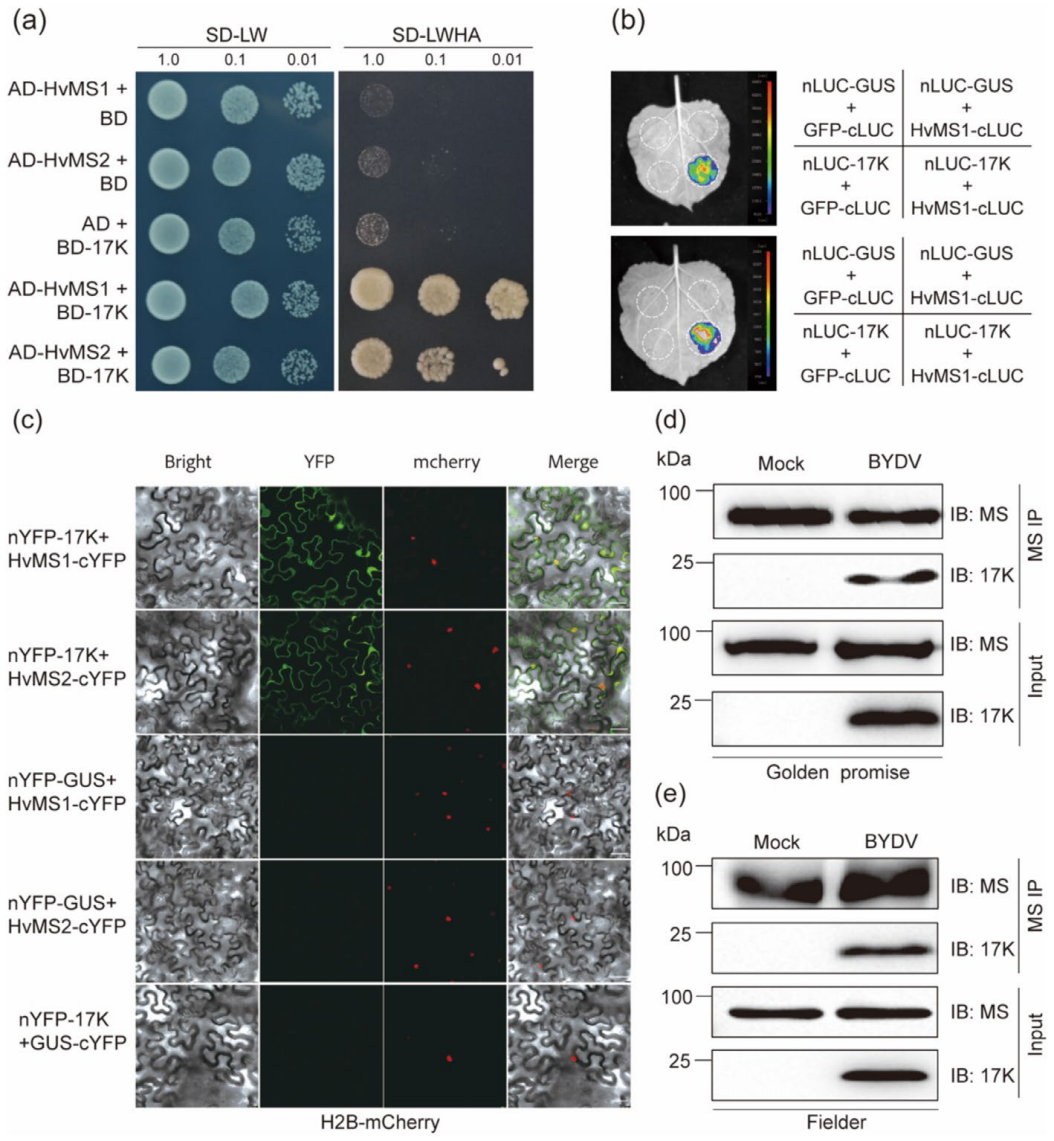
Interaction of BYDV 17K VSR with barley and wheat MS protein. (a–d) Interaction of BYDV 17K with barley HvMS1/HvMS2 revealed by Y2H (a), SLC (b), BiFC (c), and Co‐IP (d) assays. The negative controls were empty AD and BD vectors (a), nLUC‐GUS and GFP‐cLUC (b), and GUS‐nYFP and GUS‐cYFP (c). In (c), a H2B‐mCherry fusion protein was simultaneously expressed as a nuclear marker. In (d), immunoprecipitates (IP) were prepared using the total proteins extracted from mock control or BYDV infected barley plants (cv. Golden Promise) with the anti‐MS antibody developed in this study. BYDV 17K was found in the IP products derived from virus infected plants (+BYDV) but not those from mock controls. (e) Co‐IP assays showing the interaction of BYDV 17K with wheat TaMS1/TaMS2. These assays were conducted as in (d), except that the plants used were common wheat (cv. Fielder). Scale bar, 40 μm. The results presented were representative of three independent experiments.

Subsequently, we found that both HvMS1 and HvMS2 interacted with BYDV 17K in Y2H assays (Figure 1a). These interactions were verified using bimolecular fluorescence complementation (BiFC) and SLC assays (Figure 1b,c). As anticipated, six common wheat MS members, i.e., TaMS1A, TaMS1B, TaMS1D, TaMS2A, TaMS2B and TaMS2D, also interacted with 17K in SLC assays (Figure S2a). To confirm the occurrence of such interaction in virus infected barley and wheat cells, we performed Co‐IP assays using total proteins extracted from the plants inoculated with BYDV‐carrying or virus‐free aphids. The result showed that 17K could be reproducibly detected in the immunoprecipitates prepared using the MS specific antibody with BYDV‐infected barley and wheat plants, but no 17K was found in the mock controls by Co‐IP (Figure 1d,e). Collectively, the above data suggest that the MS protein can interact with BYDV 17K in both monocot and dicot plants, which stimulated us to further investigate the role of MS protein in plant virus‐host interactions.

### 2.2 Reducing *MS* Gene Expression Compromises Barley and Wheat Defence Against BYDV Infection

**FIGURE 2:**
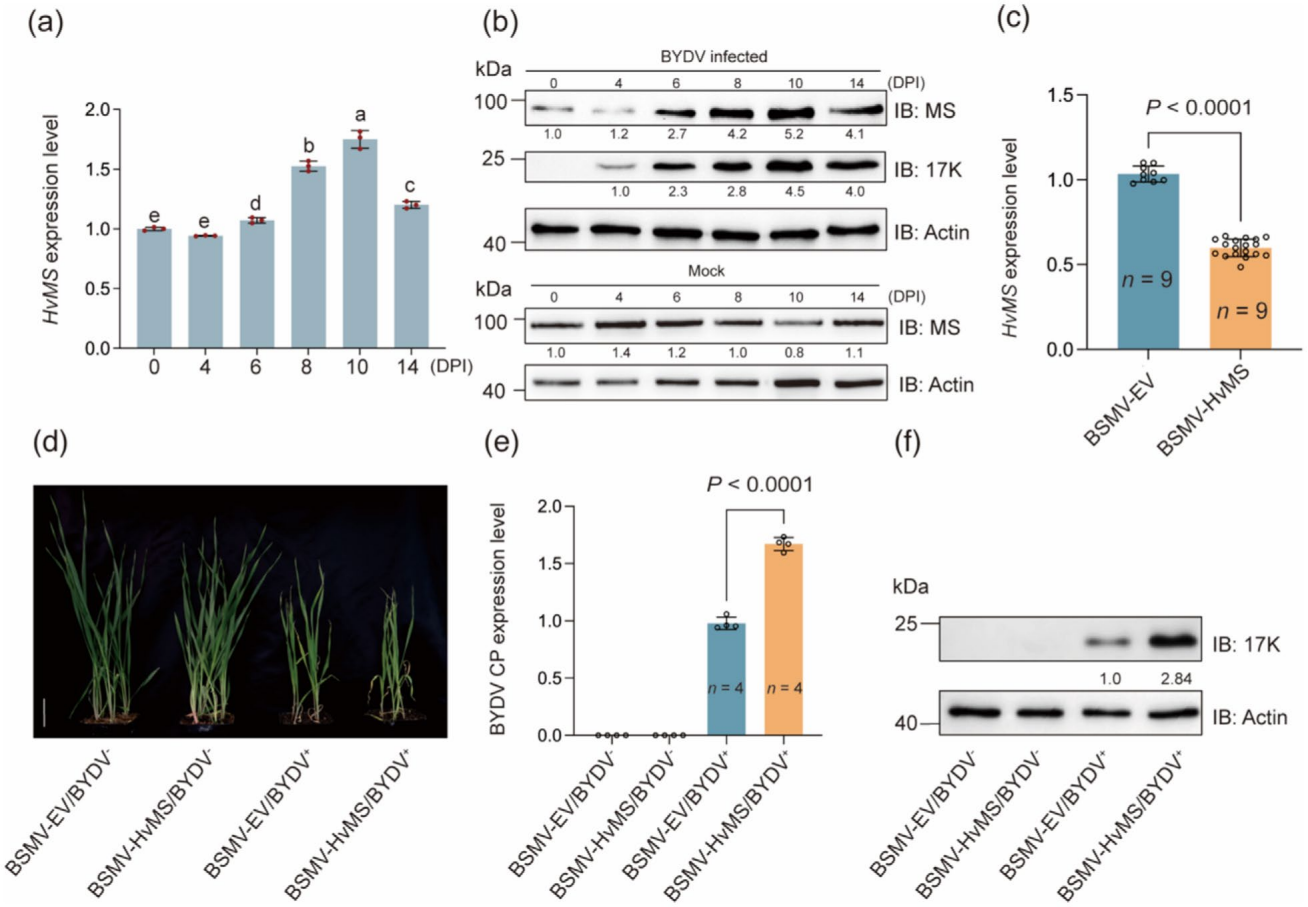
Weakening of anti‐BYDV defence by BSMV mediated silencing of *HvMS*. (a, b) Changes of *HvMS* transcript level (a) and HvMS protein abundance (b) at different days post inoculation (DPI) of BYDV in barley detected by RT‐qPCR and immunoblotting assays, respectively. (c) Significant decrease of *HvMS* gene expression by BSMV mediated gene silencing. BSMV‐EV denotes empty vector control, whereas BSMV‐HvMS represents the recombinant virus used for silencing both *HvMS1* and *HvMS2*. (d) Comparison of overall disease symptoms and plant height among the four groups of barley plants without or with *HvMS* silencing and plus or minus BYDV infection. The barley plants were pre‐infected by BSMV‐EV or BSMV‐HvMS, followed by inoculation with BYDV‐free or viruliferous aphids. (e, f) Evaluation of BYDV coat protein gene (*CP*) transcript level by RT‐qPCR assays (e) and BYDV 17K protein abundance by immunoblotting assays (f) among the four groups of plants shown in (d). In (a and c), the primers used recognised both *HvMS1* and *HvMS2*. In (b), the anti‐MS polyclonal antibody could detect the MS protein in both monocot and dicot plants (Figure S1). Detection of Actin protein as a loading control (b and f). The numerical values shown (a, c and e) were means ± SD each calculated with the data of three or more biological replicates. Statistical analysis was performed using Student's *t*‐test (c and e) or LSD pairwise multiple comparison tests (a), with different letters indicating significant differences (*p* ≤ 0.05). The intensities of the protein bands (b and f) were quantified using ImageJ (https://imagej.net/software/imagej/). Scale bar, 5 cm. The results displayed were all typical of three independent experiments.

In the barley and wheat plants infected by BYDV, *HvMS* and *TaMS* transcript levels were upregulated during the early stage, peaked at 10 days post‐inoculation (DPI), and declined at 14 DPI (Figure 2a and Figure S2b). This expression pattern was verified by examining the changes of HvMS and TaMS protein levels in BYDV‐infected plants (Figure 2b and Figure S2c). This led us to investigate whether silencing *HvMS* gene expression might affect host defence against BYDV infection using barley stripe mosaic virus‐mediated gene silencing (BSMV‐VIGS, Yuan et al. 2011). Compared with the control plants inoculated with empty BSMV vector (BSMV‐EV), *HvMS* transcript level was significantly decreased in the barley plants infected with BSMV‐HvMS designed to specifically silence both *HvMS1* and *HvMS2* (Figure 2c).

The barley plants infected with BSMV‐EV or BSMV‐HvMS were subsequently treated with virus‐free or BYDV‐carrying aphids. According to plant height recorded at 21 DPI of BYDV (Figure 2d), the plants pre‐infected by BSMV‐HvMS exhibited a higher susceptibility to BYDV infection compared to the individuals pre‐infected with BSMV‐EV. The expression level of BYDV coat protein (CP) gene was significantly higher in the *HvMS* gene‐silenced plants than in the controls without *HvMS* silencing (Figure 2e). Consistently, BYDV 17K protein level was also substantially higher in the former type of plants (Figure 2f). These results indicate that silencing *HvMS* expression weakens barley defence against BYDV infection.

**FIGURE 3:**
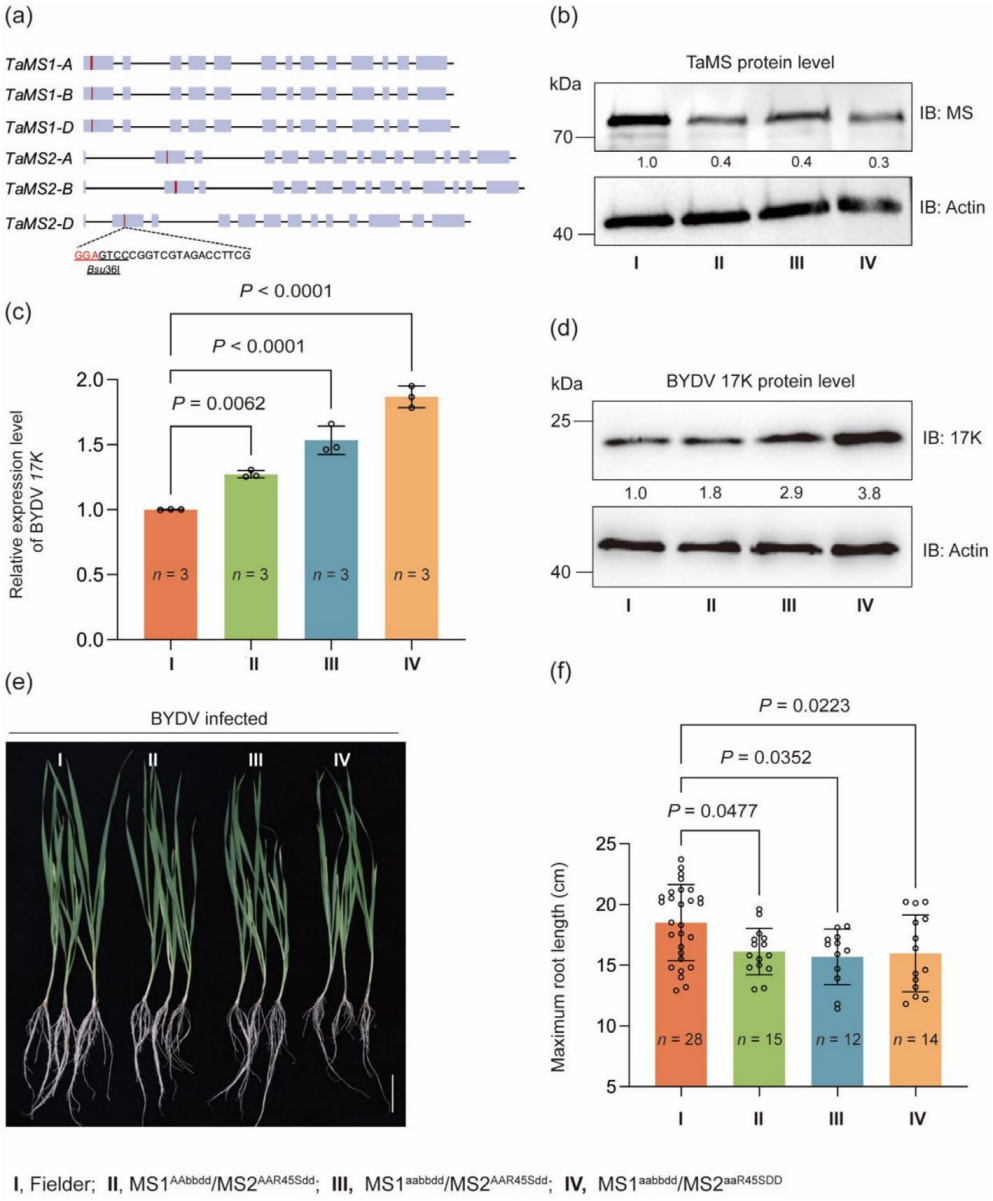
Effects of mutating *TaMS1* and *TaMS2* homoeologs by genome editing on BYDV infection in common wheat. (a) CRISPR/Cas9 mediated editing of three *TaMS1* and three *TaMS2* homoeologs using a conserved sgRNA target site, with the PAM sequence shown in red and the *Bsu*36I restriction enzyme digestion site underlined. Three resultant mutants, i.e., MS1^AAbbdd^/MS2^AAR49Sdd^, MS1^aabbdd^/MS2^AAR49Sdd^ and MS1^aabbdd^/MS2^aaR49SDD^ (Table 1, represented by II–IV in this Figure), were used in this study. (b) Comparison of TaMS protein levels in WT Fielder (I) and the three mutants (II–IV) detected by immunoblotting using the polyclonal anti‐MS antibody. The plants were grown under normal conditions, with the samples taken and analysed at three‐leaf stage. (c) Relative BYDV *17K* transcript levels in WT Fielder and the three mutants evaluated by RT‐qPCR assays at 21 DPI, with the value obtained for Fielder set as 1 to facilitate the comparison. (d) Differences in the accumulation levels of BYDV 17K protein in WT Fielder and the three mutants examined by immunoblotting at 21 DPI. (e, f) Effects of BYDV infection on the plant morphology (e) and maximum root length (f) in WT Fielder and the three mutants. The plants were grown hydroponically, with the samples collected and analysed at 21 DPI. The TaMS (b) and 17K (d) band intensities were quantified using ImageJ. The numerical values shown in (c) and (f) were means ± SD each calculated with the data of three or more biological replicates. Statistical analysis was performed using Student's *t*‐test. Scale bar, 2 cm. The results shown were representative of three independent experiments.

For investigating the effects of wheat *MS* genes on host defence against BYDV infection, we generated 193 single, double, triple, quadruple and quintuple mutants for the six *TaMS1* and *TaMS2* members in the spring wheat cultivars Fielder using CRISPR/Cas9 mediated genome editing (Figure 3a). The sgRNA target site (Figure 3a) was conserved among the two sets of *TaMS* homoeologs that were located on wheat group 4 and 5 chromosomes, respectively (Table S1). *TaMS1* and *TaMS2* sextuple mutants were not obtained despite of analysing more than 100 T_0_ transformants and their progenies. This may be caused by the fact that complete loss of *MS* gene function is lethal in plants (Escaray et al. 2022; Gonzalez and Vera 2019; Ju et al. 2020). We thus chose one quadruple (MS1^AAbbdd^/MS2^AAR49Sdd^) and two quintuple (MS1^aabbdd^/MS2^AAR49Sdd^ and MS1^aabbdd^/MS2^aaR49SDD^) homozygous and transgene‐free mutants for further experiments, because they carried knockout (KO) mutations (caused by premature stop codons in coding sequence) in at least three *TaMS* members compared to WT Fielder (MS1^AABBDD^/MS2^AABBDD^) (Table 1). Moreover, the R49S substitution in TaMS2B was likely a functionally disruptive alteration based on critical location of R49 in the protein structure modelled by AlphaFold 3; this possibility was further supported by the prediction result using the PPVED software (Gou et al. 2022), as well as those by the PROVEAN and DUTE programs (Figure S3a,b). Compared to Fielder, TaMS protein accumulation level in the three mutants was reduced by 60%–70% (Figure 3b), but the mutants did not vary significantly from Fielder in plant growth under normal greenhouse conditions (Figure S3c).

**TABLE 1:** Genotypes of the three homozygous mutants of *TaMS1/2* genes developed using CRISPR/Cas9 technology.

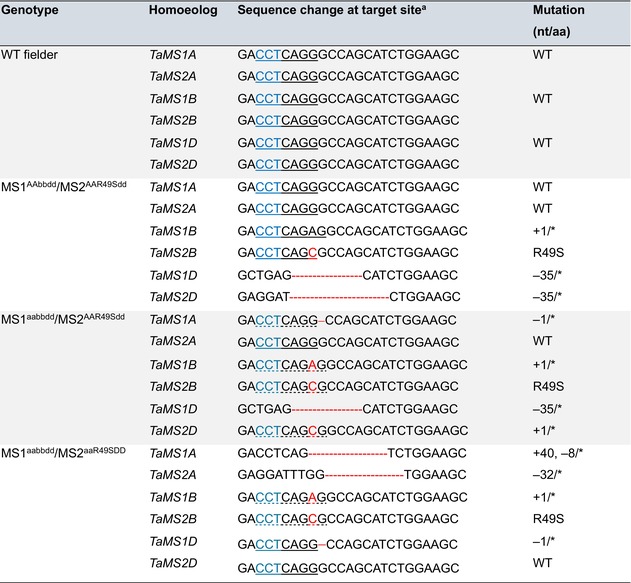

*Note:* * indicate premature stop codon mutation.

^a^ Blue letters represent PAM. Sequence changes are marked in red. The underlined sequence indicates restriction enzyme digestion site of *Bsu*36I (CCTNAGG), which is disrupted in some mutant homoeologs due to nucleotide changes (dashed line).

When inoculated with BYDV, the three mutants were more susceptible to virus infection because BYDV *17K* transcript and protein product levels were substantially higher in the mutants than in the control Fielder when examined at 21 DPI (Figure 3c,d); the average root lengths of the three mutants were also significantly shorter than that of Fielder (Figure 3e,f). When examined at the grain‐filling stage, the three mutants displayed increasingly more severe yellow dwarf symptoms compared to Fielder that was similarly infected with BYDV (Figure S3d). Note that MS1^aabbdd^/MS2^AAR49Sdd^ and MS1^aabbdd^/MS2^aaR49SDD^ plants exhibited increasingly stronger BYDV *17K* expression and more severe inhibition of root growth than did MS1^AAbbdd^/MS2^AAR49Sdd^ (Figure 3c–f), which was associated with the knockout of *TaMS1A* (MS1^aabbdd^/MS2^AAR49Sdd^) or both *TaMS1A* and *TaMS2A* (MS1^aabbdd^/MS2^aaR49SDD^). Using publicly available transcriptome data, we found that *TaMS1A* tended to be more highly expressed than its homoeologs *TaMS1B* and *TaMS1D*, with *TaMS2A* being significantly more highly expressed than its homoeologs *TaMS2B* and *TaMS2D* (Figure S3e,f).

### 2.3 Overexpressing *HvMS1* Enhances Wheat Resistance Against BYDV and BSMV

**FIGURE 4:**
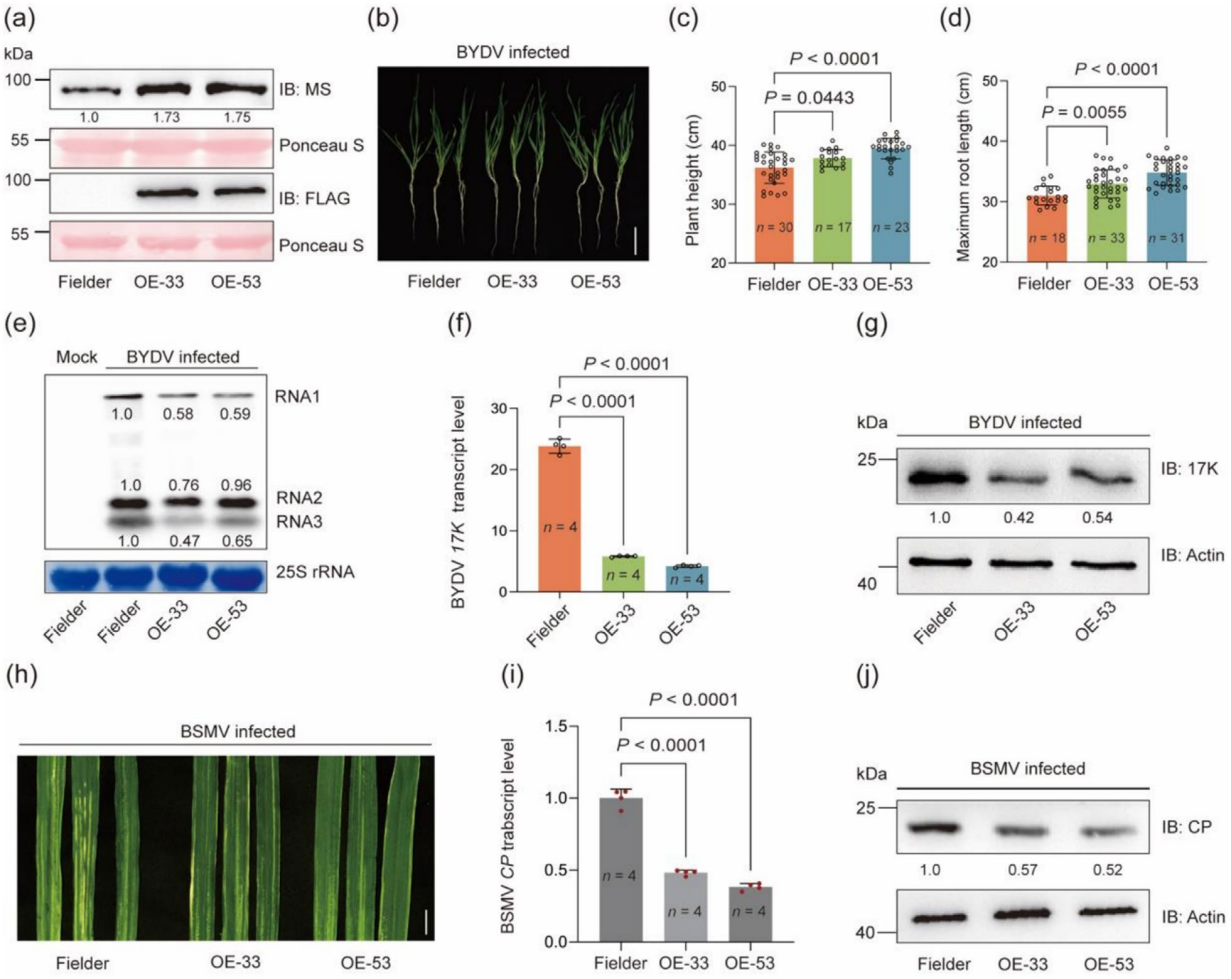
Enhancement of common wheat antiviral defence by overexpressing *HvMS1*. (a) Increased total MS protein abundance and expression of FLAG‐tagged HvMS1 in two common wheat OE lines (OE‐33 and OE‐53). Ponceau S staining of Rubisco large subunit served as a loading control. (b–d) Overall growth morphology (b), plant height (c), and maximum root length (d) of WT Fielder and two OE lines assessed at 21 DPI of BYDV. Scale bar, 3 cm. Hydroponically cultured plants of the three genotypes were inoculated with BYDV at the two‐leaf stage. (e–g) The three types of plants shown in (b) were examined for differences in the accumulation of BYDV subgenomic RNAs (RNA1, RNA2, and RNA3) (e), *17K* transcript level (f), and 17K protein abundance (g) by RNA blotting, RT‐qPCR or immunoblotting assays. (h) BSMV induced stripe mosaic symptoms in the leaves of WT Fielder and two OE lines recorded at 7 DPI. Scale bar, 1 cm. (i, j) The three types of plants shown in (h) were analysed for the transcript level (i) and protein abundance (j) of BSMV coat protein gene (*CP*) by RT‐qPCR or immunoblotting assays. In (b), hybridization of 25S rRNA served as a loading control. In (g) and (j), immunodetection of wheat Actin protein served as a loading control. In (a, g and j), protein band intensities were quantified using ImageJ. The numerical values shown in (c, d, f and i) were means ± SD each determined with the data of three or more biological replicates (*n*), with statistical analysis performed using Student's *t*‐test. The results presented were representative of three independent experiments.

The forgoing data prompted us to investigate whether overexpressing MS proteins in wheat may enhance host defence against BYDV and other viruses. Toward this end, two independent and homozygous transgenic lines (OE‐33 and OE‐53), overexpressing FLAG‐tagged HvMS1 protein in Fielder (Figure 4a), were prepared through *Agrobacterium*‐mediated genetic transformation. Under normal growth conditions, OE‐33 and OE‐53 plants showed slightly decreased plant height but increased grain size and weight as well as yield level per plant (Figure S4). In BYDV infection tests, OE‐33 and OE‐53 plants exhibited enhanced defence against BYDV compared to the control Fielder. At 21 DPI, the plant height and root length of OE plants were significantly higher than those of Fielder (Figure 4b–d). RNA blotting assays revealed that the accumulation of viral RNAs (RNA1‐3) was considerably reduced in the OE plants than in the controls (Figure 4e). In line with these observations, the abundance of BYDV *17K* transcript and protein product was strongly decreased in OE‐33 and OE‐53 compared to Fielder (Figure 4f,g).

We further tested whether OE‐33 and OE‐53 may also show improved defence against BSMV, another wheat‐infecting pathogen with a tripartite RNA genome (Jackson et al. 2009). BSMV was mechanically inoculated to the OE and Fielder seedlings at the three‐leaf stage. At seven DPI, the chlorotic and mosaic symptoms displayed by the leaves of OE plants were much less severe compared to those shown by the control leaves (Figure 4h). Consistently, the levels of BSMV *CP* transcript and protein product were clearly lower in OE‐33 and OE‐53 relative to Fielder (Figure 4i,j). Together, these data indicate that overexpressing *HvMS1* elevates wheat defence against both BYDV and BSMV.

### 2.4 *NbMS* Positively Regulates Antiviral Defence to Diverse RNA and DNA Viruses

**FIGURE 5:**
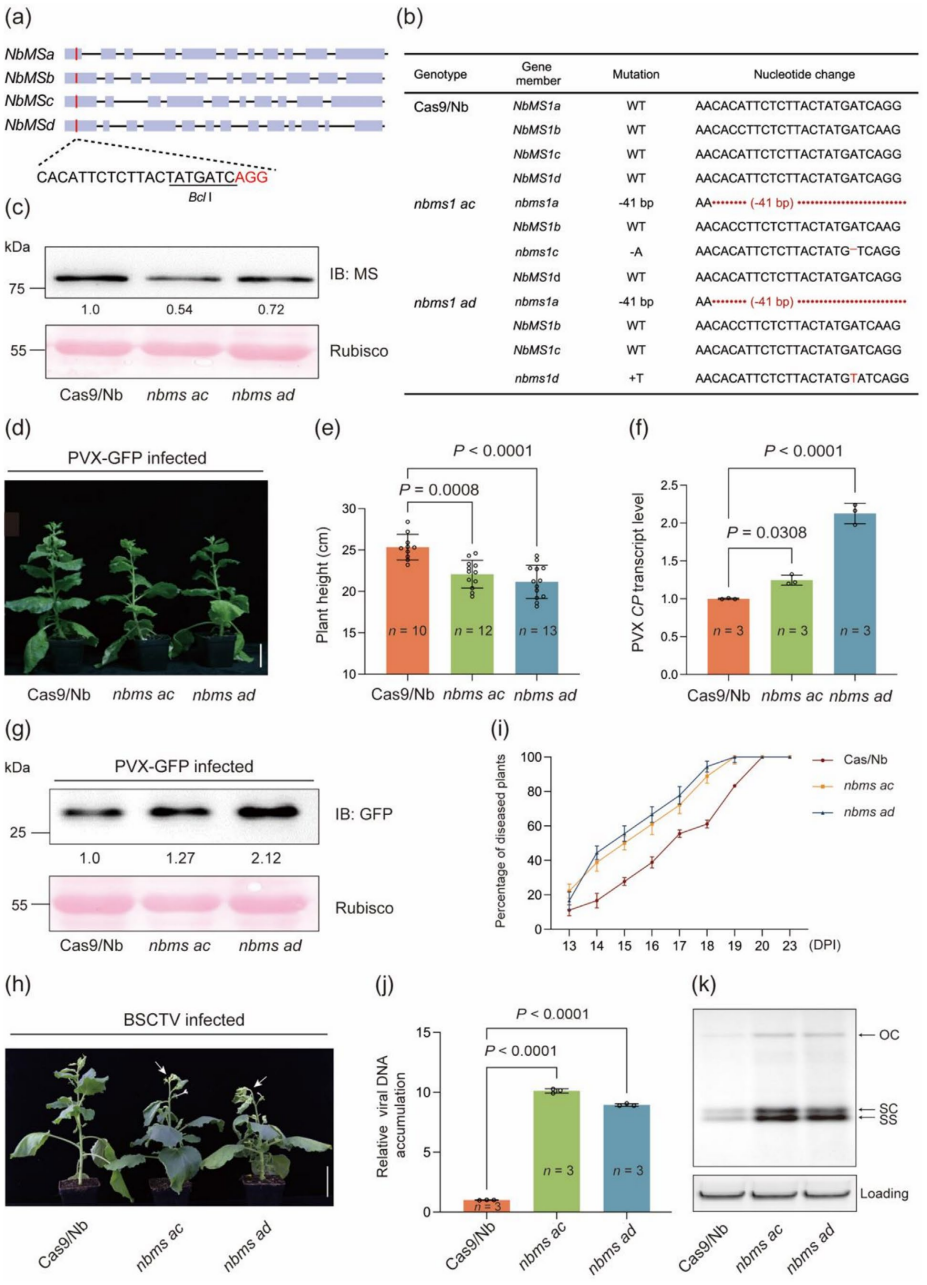
Decreased host defence to PVX or BSCTV infection in the genome editing mutants of four *NbMS* gene members. (a) Schematic presentation of the sgRNA and its target site in the four *NbMS* members (*NbMSa*—*NbMSd*) of *N. benthamiana*, with the restriction enzyme digestion site of *Bcl*I underlined and the PAM sequence shown in red. (b) Nucleotide changes (shown in red) in the two *nbms* double mutants (*nbms ac* and *nbms ad*) selected for further experiments. (c) Reduced NbMS protein levels in *nbms ac* and *nbms ad* mutants relative to that in Cas9/Nb control revealed by immunoblotting with the polyclonal anti‐MS antibody. The result was obtained with 3 weeks‐old plants grown under normal greenhouse conditions. (d, e) Morphological differences (d) and changes of plant height (e) among Cas9/Nb, *nbms ac* and *nbms ad* at 14 DPI of PVX‐GFP. (f, g) The three types of plants shown in (d) were examined for differences in the transcript level of PVX coat protein gene (*CP*) (f) and PVX mediated GFP expression (g) by RT‐qPCR or immunoblotting assays. (h) BSCTV infection symptoms in Cas9/Nb, *nbms ac* and *nbms ad* recorded at 20 DPI, with the two mutants showing a more severe curly top phenotype (arrowed) than did Cas9/Nb. (i) More rapid progression of BSCTV infection in the two mutants as compared with Cas9/Nb, which was determined by assessing the percentage of diseased plants from 13 to 23 DPI for the three genotypes (*n* = 20 plants for each genotype). (j, k) Comparison of BSCTV DNA accumulation in the three genotypes by qPCR (j) and DNA blotting (k) assays at 20 DPI. Systemically infected leaves were used in these experiments. In (j), the values obtained for the two mutants were normalised to that of Cas9/Nb, which was set as 1 to facilitate the comparison. In (k), the positions of open circle (OC), supercoiled (SC), and single‐stranded (SS) DNAs of BSCTV are indicated, with ethidium bromide‐stained genomic DNA served as a loading control. The MS protein bands in (c) and those of GFP in (g) were quantified using ImageJ. The values were means ± SD of at least 10 different plants (e) or three biological replicates (f and j) for each genotype. Statistical analysis was performed using Student's *t*‐test. Scale bar, 5 cm. The results depicted were reproducible in three independent experiments.

Based on the different sets of data presented above, we hypothesise that *MS* may positively regulate plant defence against diverse viruses. To evaluate this hypothesis, we use *N. benthamiana* as an experimental host because it can be infected by many types of RNA and DNA viruses (Bally et al. 2018). Using TRV‐mediated genome editing with Cas9 carrying *N. benthamiana* (Cas9/Nb) as recipient (Ellison et al. 2020), we developed two double mutants for four *NbMS* members (*NbMSa*—*NbMSd*) with a target site conserved among them (Figure 5a). The four members were chosen for mutant development because their deduced proteins showed comparatively high similarities (> 92%) to HvMS1 (Table S1). The two mutants carried KO mutations in either *NbMSa* and *NbMSc* (*nbms ac*) or *NbMSa* and *NbMSd* (*nbms ad*), with the level of NbMS proteins reduced by approximately 28% (*nbms ad*) or 46% (*nbms ac*) (Figure 5b,c). The *nbms ac* and *nbms ad* plants did not differ significantly from Cas9/Nb controls under normal greenhouse conditions (Figure S5a). GFP‐tagged potato virus X (PVX‐GFP), GFP expressing tobacco rattle virus (TRV‐GFP), and beet severe curly top virus (BSCTV), which carry monopartite single‐stranded RNA, bipartite single‐stranded RNA, or monopartite single‐stranded DNA genome (Macfarlane 2010; Verchot‐Lubicz et al. 2007; Zhang et al. 2011), were used as representative RNA and DNA viruses to infect *nbms ac*, *nbms ad*, and Cas9/Nb, respectively. When infected by PVX‐GFP (Figure 5d), disease symptoms were more severe in *nbms ac* and *nbms ad* plants than in Cas9/Nb controls, with the mutant plants being significantly dwarfed than the controls at 7 DPI (Figure 5d,e). The level of PVX *CP* transcripts was significantly higher in the two mutants compared to that in Cas9/Nb (Figure 5f). PVX mediated GFP expression, as revealed by immunoblotting, was also much stronger in the two mutants than in Cas9/Nb (Figure 5g). The two mutants were further found to be more susceptible to TRV‐GFP infection than Cas9/Nb (Figure S5b–e). In the infection with BSCTV, disease progression, as evidenced by an increase in the percentage of diseased plants (Figure 5h), was obviously more rapid in *nbms ac* and *nbms ad* than in Cas9/Nb. The curly top disease phenotype was stronger in the two mutants compared to Cas9/Nb (Figure 5i). The level of viral DNA accumulation, revealed using qPCR and DNA blotting assays (Figure 5j,k), was apparently higher in the two mutants than in Cas9/Nb.

**FIGURE 6:**
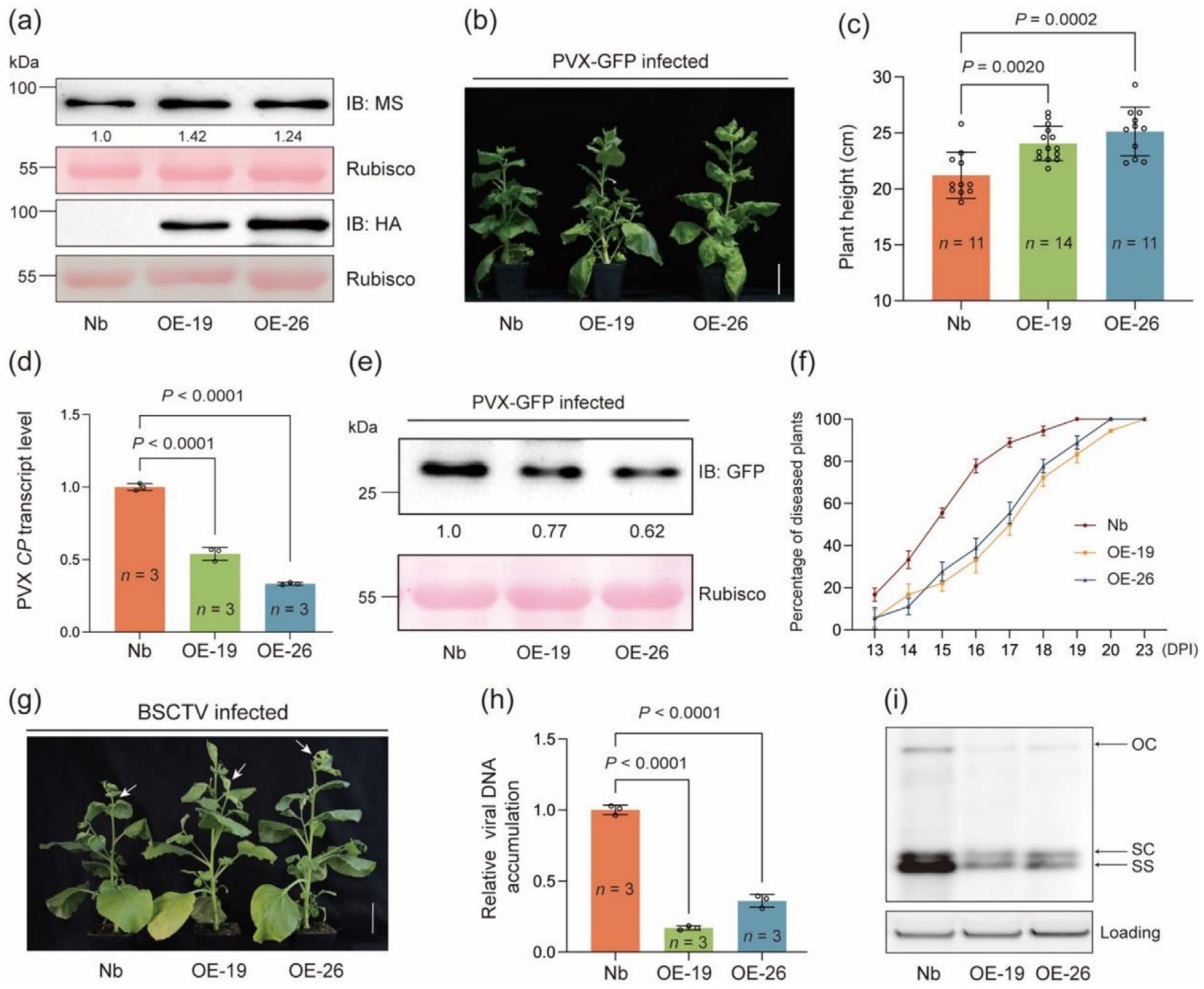
Improvement of antiviral defence by overexpressing *HvMS1* in *N. benthamiana*. (a) Elevated total MS protein abundance and expression of HA‐tagged HvMS1 in two *N. benthamiana* OE lines (OE‐19 and OE‐26). Ponceau S staining of Rubisco large subunit served as a loading control. (b, c) Morphological differences (b) and changes of plant height (c) in *N. benthamiana* (Nb, as WT control), OE‐19 and OE‐26 at 14 DPI of PVX‐GFP. (d, e) The three types of plants shown in (b) were examined for differences in the transcript level of PVX coat protein gene (*CP*) (d) and PVX mediated GFP expression (e). (f) Less rapid progression of BSCTV infection in the two OE lines compared with Nb, which was determined by calculating the percentage of diseased plants from 13 to 23 DPI for the three genotypes (*n* = 20 plants for each genotype). (g) BSCTV infection symptoms in Nb, OE‐19 and OE‐26 photographed at 20 DPI, with the curly top phenotype substantially attenuated in the two OE lines relative to that shown by Nb. (h, i) Comparison of BSCTV DNA accumulation in the three genotypes by qPCR (h) and DNA blotting (i) assays at 20 DPI. Systemically infected leaves were employed in these experiments. In (h), the values obtained for the two OE lines were normalised to that of Nb (set as 1 to facilitate the comparison). In (i), the positions of open circle (OC), supercoiled (SC), and single‐stranded (SS) DNAs of BSCTV are indicated, with ethidium bromide‐stained genomic DNA served as a loading control. The MS protein bands in (a) and those of GFP in (e) were quantified by ImageJ. The numerical values were means ± SD of at least 10 different plants (c) or three biological replicates (d and h) for each genotype. Statistical analysis was performed using Student's *t*‐test. Scale bar, 5 cm. The results shown were highly similar in three independent experiments.

We next developed two *N. benthamiana* transgenic lines overexpressing HA‐tagged HvMS1 (OE‐19 and OE‐26, Figure 6a) and assessed their responses to PVX‐GFP, TRV‐GFP or BSCTV. In the absence of viral infection, OE‐19 and OE‐26 plants grew similarly to WT controls in the greenhouse (Figure S6a). When infected by PVX‐GFP, OE‐19 and OE‐26 displayed milder disease symptoms (as indicated by differences in plant height, Figure 6b,c) and accumulated fewer viral *CP* transcripts compared with WT *N. benthamiana* (Figure 6d). The level of PVX‐mediated GFP expression was also lower in OE‐19 and OE‐26 (Figure 6e). Similar results were obtained when OE‐19, OE‐26 and WT control were compared for their reactions to TRV‐GFP infection (Figure S6b–e). In the infection assays conducted with BSCTV, disease progression was delayed, and the curly top phenotype was milder in OE‐19 and OE‐26 compared to WT control (Figure 6f,g). Viral DNA accumulated to a much lower level in the OE lines than in WT control (Figure 6h,i). These results, together with those obtained using *nbms ac* and *nbms ad* mutants (Figure 5 and Figure S5), suggest that *NbMS* is a positive regulator of antiviral defence against both plant RNA and DNA viruses.

### 2.5 Plant MS Protein Can Interact With the VSRs of Diverse RNA and DNA Viruses and Suppresses Their Anti‐Gene Silencing Function

**FIGURE 7:**
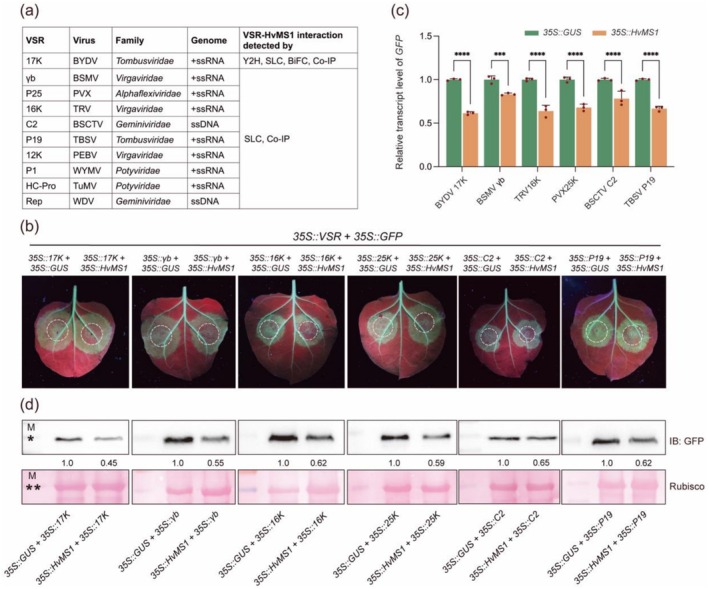
Inhibition of the anti‐gene silencing function of VSRs by HvMS1. (a) List of the 10 VSRs and corresponding viruses, which belong to five viral families and have positive‐sense single‐stranded RNA (+ssRNA) or ssDNA genome. The ten VSRs were each found to interact with HvMS1 by at least two different types of protein interaction assays. (b) Functional assays demonstrating inhibition of the anti‐gene silencing function of VSRs by HvMS1. These assays were conducted in the 16c *N. benthamiana* line that exhibits constitutive gene silencing activity. *Agrobacterium* strains carrying the *35S::GUS* or *35S::HvMS1* construct were first infiltrated into the left and right halves of the leaves of 16c tobacco, respectively. At 24 h later, *Agrobacterium* strains carrying the *35S::GFP* or *35S::VSR* construct were co‐infiltrated into the same sites, with the infiltrated areas being larger than those formed in the initial agroinfiltration (marked by dashed circles). GFP fluorescence was recorded at 3 days post the second infiltration. Transient expression of HvMS1 (in the right circled area), but not that of GUS (in the left circled area), impeded the anti‐gene silencing function of the six VSRs. (c, d) Effects of infiltrating *35S::GUS* or *35S::HvMS1* construct on the *GFP* transcript level (c) and GFP protein abundance (d) in the presence of different VSRs, as examined by RT‐qPCR or immunoblotting assays at 3 days post the second infiltration. In (c), the relative values, normalised to that obtained for *35S::GUS* (set as 1), were means ± SD of three biological replicates. In (d), GFP band intensities were quantified using ImageJ. The molecular mass standards labelled by * and ** in the marker lane (M) were 30 and 55 kDa, respectively. Statistical analysis was performed using Student's *t*‐test (****p < 0.001). The results shown were reproducible over three independent experiments.

To gain mechanistic insight into the positive regulation of antiviral defence by plant MS protein, we explored whether it may bind the VSRs of different viruses and suppress their anti‐gene silencing function. We carried out two sets of experiments using HvMS1 as a representative since it interacted with BYDV 17K VSR (Figure 1). In the first set, the VSRs used included BSMV γb (Bragg and Jackson 2004), PVX 25K (Bayne et al. 2005; Voinnet et al. 2000), TRV 16K (Ghazala et al. 2008; Martín‐Hernández and Baulcombe 2008; Martínez‐Priego et al. 2008), and BSCTV C2 (Zhang et al. 2011). The P19 protein of tomato bushy stunt virus (TBSV), whose VSR function has been well studied in the past (Lakatos et al. 2004; Silhavy et al. 2002), was used as a control. The five VSRs all interacted with HvMS1 in the SLC and Co‐IP assays performed in *N. benthamiana* (Figure 7a and Figure S7). As shown in Figure 7b, the five VSRs, as well as BYDV 17K, could all revive GFP expression in 16c tobacco, a system that has been widely employed to reveal the anti‐gene silencing effect of VSRs (Ruiz et al. 1998; Voinnet et al. 1999). However, the ability of the six VSRs to restore GFP expression in 16c tobacco was generally and significantly down‐regulated in the presence of HvMS1 expression as indicated by decreases in GFP fluorescence, transcript level, and protein accumulation (Figure 7b–d). As a control to HvMS1, transient expression of β‐glucuronidase (GUS) did not affect the six VSRs to restore GFP expression (Figure 7b–d).

In the second set of experiments, the VSRs of four additional viruses, i.e., the HC‐Pro VSR of turnip mosaic virus (Li and Wang 2018; Valli et al. 2018), the P1 VSR of wheat yellow mosaic virus (Chen et al. 2023), the putative 12K VSR of pea early‐browning virus (Escalante et al. 2024), and the Rep VSR of wheat dwarf virus (Liu et al. 2014), were found to interact with HvMS1 in both SLC and Co‐IP assays (Figure 7a and Figure S8). The four VSRs all exhibited anti‐gene silencing effects in 16c tobacco (Figure S9a); their VSR activities were also markedly reduced by HvMS1 expression but not that of GUS (Figure S9b,c). Hence, the two sets of experiments demonstrate that plant MS protein, as represented by HvMS1, is capable of interacting with the VSRs of different RNA and DNA viruses and inhibits their anti‐gene silencing function.

## 3 Discussion

In this work, we discovered the positive regulation of antiviral defence by plant MS protein. Our analysis led to the identification of a new inhibitor of plant viral VSRs, which may be employed to develop crops with broad spectrum antiviral resistance.

Prior to this work, there is no direct genetic and molecular evidence on the regulation of antiviral defence by plant MS protein, although *MS* gene expression was reported to be induced by soybean mosaic virus (SMV), citrus leprosis virus (CiLV), and potato virus Y (PVY) (Babu et al. 2008; Fesenko et al. 2021; Freitas‐Astúa et al. 2007; Spechenkova et al. 2021). Based on the VIGS result generated in barley and the genetic data obtained from analysing genome editing and OE lines of common wheat and *N. benthamiana* (Figures 2, 3, 4, 5, 6 and Figures S5 and S6), it is clear that plant MS protein positively regulates antiviral defence against diverse RNA and DNA viruses. This suggestion is consistent with apparent induction of the transcripts and proteins of *MS* genes upon BYDV infection of barley and wheat (Figure 2a,b and Figure S2b,c). Therefore, we speculate that the increased *MS* gene expression, observed in SMV and CiLV infected plants (Babu et al. 2008; Freitas‐Astúa et al. 2007), may also function to strengthen host antiviral defence.

What are the mechanisms underlying the positive regulation of antiviral defence by plant MS protein? Judging from the findings that HvMS1 interacted with the VSRs of BYDV and other nine RNA and DNA viruses examined in this work and that HvMS1 expression significantly lowered the anti‐gene silencing function of different VSRs (Figure 7 and Figures S7–S9), we deduce that inhibition of VSR function likely plays an important role in the positive contribution of plant MS protein to host antiviral defence. In line with this proposition, previous studies have identified the existence and function of several plant proteins (rgs‐CaM, ZmVDE, and ZmTGL) that interact with, and thereby disrupt the RNAi suppression function of, VSRs (Chen et al. 2017; Jeon et al. 2017; Nakahara et al. 2012; Xu et al. 2022). Thus, plant MS protein, such as HvMS1, may represent a new VSR inhibitor that contributes to the antiviral defence in both monocot (barley and wheat) and dicot (*N. benthamiana*) plants via binding and debilitating the VSRs of diverse RNA and DNA viruses.

In contrast to the insight outlined above, two recent studies suggest that enhancement of transmethylation, associated with viral infection induced upregulation of MTC genes, may be responsible for potato plants (cv. Gala) to restrict PVY proliferation (Fesenko et al. 2021; Spechenkova et al. 2021). A significant rise in the concentration of SAM was observed in the PVY resistant cultivar Gala but not the susceptible variety Chicago (Spechenkova et al. 2021). Because SAM is a universal methyl donor in cellular methylation reactions and an important precursor for ethylene biosynthesis in plants (Mäkinen and De 2019; Roje 2006), the researchers hypothesised that increased SAM accumulation may lead to PVY resistance in Gala through stabilisation of vsiRNAs by SAM‐dependent HEN1 methyltransferase as well as by potentially elevated ethylene content (Fesenko et al. 2021; Spechenkova et al. 2021). The latter proposition is additionally supported by the observation that efficient ethylene biosynthesis and signalling are beneficial for watermelon to resist the infection by Cucumber green mottle mosaic virus (Liu et al. 2024). From the studies discussed above, it will be interesting to check whether SAM and ethylene contents may increase in the *HvMS1* OE lines. Further investigations along this direction may enable a more complete elucidation of the molecular and biochemical mechanisms underlying MS mediated positive regulation of antiviral defence in plants.

The finding that HvMS1 could interact with the VSRs of ten different plant RNA and DNA viruses is intriguing considering that these VSRs generally exhibit low amino acid sequence identities (Table S2). The rgs‐CaM protein could also interact with divergent VSRs like potyviral HC‐Pro and cucumoviral 2b (Nakahara et al. 2012). Structural analysis suggests that rgs‐CaM binds the dsRNA domain that is present in many VSRs (Nakahara et al. 2012; Tadamura et al. 2012). However, rgs‐CaM did not interact with the P19 VSR of TBSV, probably due to the fact that P19 did not bind to long dsRNA directly (Lakatos et al. 2004; Silhavy et al. 2002). In contrast, our work uncovered that HvMS1 could interact with P19 in both SLC and Co‐IP assays (Figure S7). Thus, the process involved in VSR binding by HvMS1 may be more complex and differ from that used by rgs‐CaM. Our preliminary investigation showed that both the putative N‐ and C‐terminal domains of HvMS1 interreacted with four representative VSRs in Y2H assays (Figure S10). We then modelled electrostatic potential for the HvMS1 structure predicted with AlphaFold 3 (Abramson et al. 2024), and found a positive electrostatic patch, which involves many basic amino acid residues located in the N‐ or C‐terminal domains of HvMS1 (Figure S11a,b). Interestingly, the predicted structures of the 10 VSRs all possess the surface with prominent negative electrostatic potential, which could interact with the positive electrostatic patch of HvMS1 in modelled HvMS1‐VSR complex (Figure S11c). Therefore, HvMS1‐VSR interaction is possibly facilitated by electrostatic complementarity at protein–protein interface. This aligns with the observation that plant viral MPs, many of which are themselves VSRs, tend to have common structural segments (Atabekova et al. 2023). We are now in the process of testing the involvement of electrostatic complementarity in HvMS1‐VSR interaction. As to how plant MS protein may disrupt the anti‐gene‐silencing activity of VSRs, we speculate that binding of MS to VSRs may impair their ability to suppress the enzymes or other critical components required for vsiRNA production and function.

As climate change intensifies, the incidence and severity of plant viral diseases, including those elicited by BYDVs, are projected to increase globally (Jones and Naidu 2019; Singh et al. 2023; Tatineni and Hein 2023; Trebicki 2020). The development of crop cultivars with strong antiviral BSR is highly desired for sustainable control of plant viruses. From the data and insight generated in this work, plant MS protein may represent a valuable target for engineering antiviral BSR in both monocot and dicot crops. Notably, the rice *MS1* gene (also named as *METS1*) has been found to positively regulate resistance against blast disease caused by the fungal pathogen *Magnaporthe grisea* (Zhai et al. 2022). In *Arabidopsis*, *MS1* contributes to resistance against the bacterial pathogen *Pseudomonas syringae* DC3000 (Escaray et al. 2022; Gonzalez and Vera 2019). These studies plus the work here indicate that plant MS protein has a broader role in host defence against multiple types of phytopathogens. It is worth noting that the wheat OE lines expressing HvMS1 showed improved agronomic traits (e.g., seed size and yield per plant) (Figure S4). This raises the possibility that overexpressing plant MS protein may confer resistance to viral, fungal, and bacterial pathogens as well as improved yield performance. To evaluate this possibility, we are now in the process of testing the transgenic wheat and *N. benthamiana* lines overexpressing *HvMS1* with more microbial pathogens.

Overall, this work reveals the promotion of antiviral defence by MS protein in both monocot and dicot plants. Further studies and appropriate genetic engineering of plant MS protein, which is achievable using AI assisted protein/mRNA modification and innovative genome editing technologies (Fei et al. 2025; Qin et al. 2025; Zhang et al. 2025; Zhao et al. 2024), may facilitate the development of crop varieties with superior BSR against a wide range of phytopathogens including both RNA and DNA viruses.

## 4 Materials and Methods

### 4.1 Plant Materials and Growth Conditions, BYDV Inoculation Conditions, VSR Coding Sequence, PCR Primers, DNA Constructs and Antibodies

Barley (Golden Promise), *Arabidopsis* (Col‐0), wheat (Fielder) and tobacco (*N. benthamiana*) plants were grown under controlled environments (16 h light/8 h dark photoperiod; 22/20°C day/night temperatures; 60% relative humidity) in greenhouse/growth chambers. BYDV‐GAV was inoculated to barley or wheat plants as reported in previous studies (Jin et al. 2020, 2022; Wang et al. 2024). The 17K VSR coding sequence was cloned from BYDV‐GAV genome (Jin et al. 2020). The coding sequences for the remaining nine VSRs, as listed in Figure 7a and Table S2, were all synthesised commercially (Shenggong, shanghai, China). The PCR primer sequences, DNA constructs, and antibodies used in this work are provided in Tables [Link], [Link], [Link].

### 4.2 IP‐MS and Co‐IP Assays

The IP‐MS experiment that led to the identification of 17K interacting proteins (including AtMS1 studied in this work) was described previously (Wang et al. 2024). Co‐IP assays were performed to detect protein–protein interactions in various *Arabidopsis*, tobacco, barley, or wheat samples essentially as described in our previous studies (Jin et al. 2020, 2022; Wang et al. 2024). Briefly, Total proteins were extracted from desired plant samples using lysis buffer (50 mM Tris–HCl pH 7.6, 150 mM NaCl, 5 mM MgCl_2_, 10% (v/v) glycerol, 0.1% (v/v) NP‐40, 0.5 mM DTT, 1× protease inhibitor cocktail, 1 mM PMSF), followed by centrifugation for 15 min (18 200 rpm) at 4°C. The resultant supernatants were filtered through Miracloth (Merck Millipore) and incubated for 4 h or overnight at 4°C with the following magnetic beads: GFP‐Nanoab‐Magnetic Beads (LABLEAD, GNM‐25‐1000) for detecting the interaction between 17K‐GFP and AtMS1; HA‐Nanoab‐Magnetic Beads (LABLEAD, HNM‐25‐1000) and DYKDDDDK‐Nanoab‐Magnetic Beads (LABLEAD, FM0500) for detecting the interaction between viral VSR proteins and HvMS1; DynaGreen Protein A/G Magnetic Beads (Thermo Fisher Scientific, 80106G) cross‐linked with HvMS1 antibody for detecting the interaction between 17K and HvMS1 or TaMS1. Thereafter, the beads were collected and washed four times with washing buffer. The protein complexes were eluted by boiling the beads in 2× SDS‐PAGE sample buffer for 5 min, separated using 12% SDS‐PAGE, and subjected to immunoblotting with selected antibodies (Table S5).

### 4.3 Bioinformatic Identification of Plant *MS* Genes

AtMS1 amino acid sequence was used as a query to search the *Arabidopsis* Information Resource (https://www.arabidopsis.org/), Ensembl Plants (https://plants.ensembl.org/index.html), and SOL Genomics Network (https://solgenomics.net/) databases. The deduced amino acid sequences of *MS* genes from representative species ( *A. thaliana* , *N. benthamiana*, *H. vulgare* , *T. aestivum* , *O. sativa* , *Z. mays* and *C. reinhardtii* ) were compared for identities and similarities using the software MatGAT (Campanella et al. 2003), with the molecular mass of the deduced MS proteins calculated in the Expasy website (https://web.expasy.org/compute_pi/) (Table S1).

### 4.4 RNA Extraction and RT‐qPCR Assays

Total RNAs were isolated from plant tissues using TRIzol Reagent (Sangon, B511311) following the manufacturer's protocol. First‐strand cDNA synthesis was performed with All‐In‐One 5× RT MasterMix (ABM, G592), which included integrated gDNA removal, according to the manufacturer's instructions. RT‐qPCR assays were conducted using ChamQ Universal SYBR qPCR Master Mix (Vazyme, Q711‐02) under optimised conditions. In the RT‐qPCR assays conducted with barley or *N. benthamiana* samples, amplification of the actin gene was used as an internal reference. In the assays performed with common wheat samples, amplification of an α‐tubulin gene was employed as an internal control. Primer pairs specific for reference and target genes were listed in Table S3.

### 4.5 Y2H Assays

The yeast strain AH109 was co‐transformed with pGBKT7‐17K and either the pGADT7 empty vector control or pGADT7‐MS genes using the Clontech yeast transformation protocol. Transformants were grown on SD‐Leu‐Trp dropout medium and selected on SD‐Leu‐Trp‐His medium plates. All Y2H experiments were repeated three times independently.

### 4.6 SLC Assays

These assays were carried out following established protocols (Chen et al. 2008; Jin et al. 2020). The pCAMBIA1300‐nLUC and pCAMBIA1300‐cLUC vectors were utilised to express N‐terminal and C‐terminal luciferase fusions of candidate interacting partners, respectively. All constructs were introduced into *A. tumefaciens* strain GV3101 and subsequently delivered into *N. benthamiana* leaves via agroinfiltration. Leaf tissues were harvested 36–48 h post‐infiltration (hpi), and luciferase activity was quantified using a NightSHADE evo LB 985 imaging system (Berthold Technologies, Bad Wildbad, Germany).

### 4.7 BiFC Assays

Bimolecular fluorescence complementation (BiFC) assays were performed to validate protein–protein interactions (Waadt et al. 2008). The full‐length coding sequence of *MS* genes and that of *VSR* genes were cloned into pXY106 (^N^YFP) and pXY104 (^C^YFP) vectors, respectively, generating N‐terminal YFP fusions (MS‐^N^YFP) and C‐terminal YFP fusions (VSR‐^C^YFP). All constructs were introduced into *A. tumefaciens* strain GV3101. The *Agrobacterium* suspensions containing pairwise combinations of constructs were mixed at a 1:1 ratio and co‐infiltrated into *N. benthamiana* leaves using a needleless syringe. YFP fluorescence signals were visualised 36–48 hpi using a Zeiss LSM710 confocal microscope. Tobacco cell nuclei were visualised by co‐expressing the AtH2B‐mCherry nuclear marker (Wang et al. 2024).

### 4.8 Preparation of a Polyclonal Antibody Specific for Plant MS Protein

Recombinant HvMS1 protein was expressed and purified as previously described (Jin et al. 2020, 2022). The coding sequence of *HvMS1* was cloned into the pET30a expression vector and transformed into *E. coli* BL21 (DE3) cells. Histidine‐tagged recombinant protein was purified via Ni‐NTA affinity chromatography. Polyclonal antiserum against HvMS1 was generated by immunising four mice with purified protein via intramuscular injection. Antibody specificity was validated by immunoblotting assays, which demonstrated specific recognition of the MS protein from *N. benthamiana* (NbMS), *H. vulgare* (HvMS), and *T. aestivum* (TaMS) (Figure S1e,f).

### 4.9 Agroinoculation of Tobacco With Plant Viruses

For PVX inoculation, a recombinant PVX construct expressing mGFP5 was developed using the vector pgR106 (Lu et al. 2003), which was then introduced into *A. tumefaciens* strain GV3101. The *Agrobacterium* suspension (OD_600_ = 0.01) for expressing PVX‐GFP was infiltrated into *N. benthamiana* plants (Dalmay et al. 2000). For TRV inoculation, TRV‐GFP was introduced into *N. benthamiana* leaves as reported by Sha et al. (2014). GFP fluorescence in the plants infected by PVX‐GFP or TRV‐GFP was monitored under UV illumination. Symptom development was recorded photographically at 14–21 dpi. For BSCTV inoculation, the binary vector pCambia1300‐BSCTV was introduced into *A. tumefaciens* strain EHA105 (Ji et al. 2015). The *Agrobacterium* suspension (OD_600_ = 0.2) for expressing BSCTV was infiltrated into fully expanded leaves of five leaf‐stage *N. benthamiana* plants. Systemically infected leaves were harvested at 14 days post‐inoculation (dpi) for genomic DNA extraction and Southern blot analysis.

### 4.10 Gene Silencing Mediated by BSMV

BSMV‐VIGS was performed in barley as previously described (Wang et al. 2024; Yuan et al. 2011). In brief, a 226‐bp conserved fragment targeting *HvMS1/2* was cloned into the BSMV RNAγ vector pCa‐γbLIC via ApaI‐mediated ligation‐independent cloning to generate pCa‐γbLIC::HvMS. Tripartite BSMV components, pCaBS‐α (RNAα), pCaBS‐β (RNAβ), and pCa‐γbLIC::HvMS or empty vector (EV) control, were introduced into *A. tumefaciens* EHA105 and co‐infiltrated into *N. benthamiana* to produce infectious BSMV‐HvMSs or BSMV‐EV particles. Systemic leaf sap from tobacco was mechanically inoculated onto carborundum‐dusted leaves of two‐leaf‐stage barley (Golden Promise), with *HvMS* silencing efficiency confirmed by RT‐qPCR at 10 dpi using third fully expanded leaves. HvMS silenced plants (*n* ≥ 30/group), as well as those pre‐infected with BSMV‐EV, were then exposed to: non‐viruliferous *Schizaphis graminum* reared on BYDV‐free barley or viruliferous *S. graminum* from BYDV‐GAV infected source plants. One week later, the aphids were eliminated via imidacloprid foliar spray (2 mg/L). Plant height and accumulation levels of BYDV products (i.e., *CP* transcript and *17K* transcript/protein) were assessed at 3 weeks post‐inoculation.

### 4.11 Construction of Transgenic Wheat and Tobacco Lines Overexpressing *HvMS1*

Immature embryos of Fielder were transformed via *A. tumefaciens* ‐mediated delivery of pWMB122‐*Ubi::HvMS1‐3 × FLAG* (Ishida et al. 2015), while *N. benthamiana* leaf discs were treated with pC1300‐*35S::HvMS1‐3 × HA* (Horsch et al. 1985). T0‐T3 plants were analysed by genomic PCR, RT‐qPCR, and immunoblotting assays, with homozygous stable T3 lines propagated for studying the effects of overexpressing *HvMS1* on antiviral defence.

### 4.12 Genome Editing of *TaMS1* and *TaMS2* Homoeologous

CRISPR/Cas9 editing targeting *TaMS* homoeologs was performed as described in our previous study (Wang et al. 2024). A specific sgRNA sequence, conserved among the six *TaMS* members (Figure 3a), was cloned into the binary vector pLH3. The resulting construct was used to transform the immature embryos of Fielder. T0 transformants were genotyped using PCR‐restriction enzyme (PCR‐RE) analysis and Sanger sequencing with homoeolog‐specific primers. Through successive screening of T0–T3 generations, three stable homozygous lines, carrying KO or other types of disruptive mutations in 4–5 *TaMS* gene members (Table 1), were selected for subsequent experiments.

### 4.13 Genome Editing of *NbMS* Genes

TRV‐mediated virus‐induced genome editing (TRV‐VIGE) was employed to target four *NbMS* members (*NbMSa*—*NbMSd*, Table S1) following the established protocol (Ellison et al. 2020). A specific sgRNA sequence, targeting a conserved region of 4 *NbMS* members (Figure 5a), was designed and cloned into the pTRV‐Cr vector. The recombinant vector was introduced into *A. tumefaciens* GV3101 (OD_600_ = 0.3) for infiltration of Cas9‐overexpressing tobacco plants. Through PCR‐RE analysis and Sanger sequencing of T1–T3 plants using gene‐specific primers, two homozygous double mutant lines, *nbms ac* and *nbms ad* (Figure 5b,c), were developed for subsequent experiments.

### 4.14 RNA Blotting

RNA blotting for detecting BYDV RNA accumulation in host plants was performed as detailed by Wang et al. (2024). Total RNA samples (15 μg each), extracted from BYDV‐GAV‐infected or mock control wheat plants at 10 dpi, were separated in a 1% formaldehyde‐denatured agarose gel, followed by transfer to a Hybond^−^ *N* ^+^ nylon membrane (Cytiva, RPN303B). The membrane was hybridised with a biotinylated BYDV‐GAV‐specific probe labelled with Biotin‐11‐dUTP (Thermo, R0081). Chemiluminescent detection was performed with the BeyoECL Plus Kit (Beyotime, D3308) according to manufacturer specifications.

### 4.15 DNA Blotting

DNA blotting for evaluating BSCTV accumulation in *N. benthamiana* plants was conducted as described by Gui et al. (2022). Total DNA samples (2 μg each), extracted from tobacco leaves at 14 days post BSCTV infiltration, were electrophoresed using 0.9% agarose gel, followed by denaturation (0.5 M NaOH/1.5 M NaCl) and neutralisation (1 M Tris–HCl pH 7.4/1.5 M NaCl). The separated DNA was capillary‐transferred to Hybond^−^ *N* ^+^ membrane using 20× SSC buffer (3 M NaCl, 0.3 M trisodium citrate, pH 7.0), UV‐crosslinked, and vacuum‐baked at 80°C for 2 h. The membrane was pre‐hybridisation (65°C, 4 h) in PerfectHyb Plus Buffer (Sigma‐Aldrich, H7033), then hybridised overnight (65°C) with a biotinylated BSCTV specific probe. Post‐hybridisation washes included sequential treatments with 1× SSC/0.1% SDS (65°C, 20 min), and 0.1× SSC/0.1% SDS (65°C, 10 min). Chemiluminescent signals were recorded using the BeyoECL Plus Kit (Beyotime, D3308) in accordance with the manufacturer's protocol.

### 4.16 Inhibition of VSR Activity by Transient Expression of HvMS1

These assays were executed essentially as described by Jin et al. (2022). The coding sequences of viral VSRs, HvMS1‐3× FLAG, and β‐glucuronidase (GUS) were each cloned into the pEG100 vector, resulting in a series of 35S promoter directed transient expression constructs. The *A. tumefaciens* GV3101 strains carrying *35S::GUS* or 35S::*HvMS1‐3× FLAG* were first infiltrated into the left and right halves of the leaves of 16c tobacco, respectively. At 24 h post the initial infiltration, the *Agrobacterium* strain carrying *35S::GFP*, plus the one harbouring *35S::VSR*, were co‐infiltrated into the same sites, with the infiltrated areas being larger than those in the initial agroinfiltration. GFP fluorescence was examined at 3 days post the second infiltration using a Blak‐Ray B‐100AP UV lamp (UVP, 76005501). Inhibition of VSR activity by transiently expressed HvMS1 was first indicated by decreased GFP fluorescence in the leaf area infiltrated with 35S::*HvMS1‐3× FLAG* but not that infiltrated with *35S::GUS*, which was subsequently verified by quantifying GFP transcript and protein levels using RT‐qPCR and immunoblotting assays, respectively.

### 4.17 Bioinformatic Analysis and Protein Modelling

Plant MS homologues were searched in the NCBI database (https://www.ncbi.nlm.nih.gov/) or the Ensemble genome database (https://plants.ensembl.org/index.html). Phylogenetic analysis was conducted using plant MS amino acid sequences at the MEGA website (https://www.megasoftware.net/). The neighbour‐joining program was used to construct the consensus phylogenetic tree with complete deletion and 1000 bootstrap permutations. Protein modelling was executed using AlphaFold 3 (Abramson et al. 2024). Three‐dimensional modelling of protein electrostatics was accomplished using ChimeraX (Pettersen et al. 2021).

## Author Contributions

Liyuan You, Huaibing Jin and Daowen Wang conceived and supervised the project. Zhaohui Wang, Kunpu Zhang and Chi Zhang performed the majority of the experiments. Jin Yang, Bei Li, Huihui Bi and Lina Wang assisted in the generation and maintenance of wheat OE and genome editing mutants. Zhenghao Shi, Rui Guo, Shuai Zhang, Kaiqi Gao and Jianing Li helped with the preparation and growth of *N. benthamiana* OE and genome editing mutants. Xiaohuan Jin and Xiang Ji analysed the effects of R49S substitution in TaMS2B. Zhaohui Wang and Liyuan You wrote the original draft. Daowen Wang, Liyuan You and Huaibing Jin finalised the paper.

## Funding

This work was supported by Zhongyuan Scholar Program, 234000510002. The Shennong Laboratory, SN01‐2022‐01. The National Natural Science Foundation of China, 32401930. The Scientific and Technological Research Project of Henan Province of China, 242102111161.

## Conflicts of Interest

The authors declare no conflicts of interest.

## Supporting information


**Figure S1:** Identification of AtMS1 as an interacting protein of BYDV 17K VSR.
**Figure S2:** Detection of BYDV 17K‐TaMS interaction by SLC assays and regulation of *TaMS* expression by BYDV infection.
**Figure S3:** Potential effect of R49S substitution in TaMS2B, growth performance of three *TaMS* CRISPR mutants, and expression levels of six *TaMS* members.
**Figure S4:** Comparison of WT Fielder and two derivative HvMS1 OE lines grown under normal conditions.
**Figure S5:** Decreased host defence against TRV caused by mutating *NbMS* gene members in *N. benthamiana*.
**Figure S6:** Increased host defence against TRV conferred by overexpressing *HvMS1* in *N. benthamiana*.
**Figure S7:** Interaction of a representative plant MS protein (HvMS1) with the VSRs of five different RNA and DNA viruses.
**Figure S8:** Interaction of HvMS1 with the VSRs of four additional RNA and DNA viruses.
**Figure S9:** Impairment of the anti‐gene silencing function of four different VSRs by HvMS1.
**Figure S10:** Analysis of the structure elements of HvMS1 involved in interacting with VSRs in Y2H assays.
**Figure S11:** Modelling analysis of the interaction between HvMS1 and VSRs.

**Table S1:** Amino acid sequence identities and similarities among the MS proteins of representative plant species.

**Table S2:** Amino acid sequence identities and similarities among the 10 plant viral VSRs investigated in this study.

**Table S3:** The oligonucleotide primers used in this study.

**Table S4:** Summary of the DNA constructs used in this study.

**Table S5:** Description of the primary and secondary antibodies used in this study.

## Data Availability

The data pertinent to this work are all described in the main paper and the Supporting Information.
