# Supplementary material for: Methionine Synthase Positively Regulates Plant Defence to Both RNA and DNA Viruses and Is Useful for Developing Broad‐Spectrum Antiviral Resistance in Crops
Source: Plant Biotechnol J. 2026 Feb 23;24(6):3821–39. doi: 10.1111/pbi.70618 (PMC13205620; doi:10.1111/pbi.70618)
Supplement: Supplementary file 1 — Figure S1: Identification of AtMS1 as an interacting protein of BYDV 17K VSR. Figure S2: Detection of BYDV 17K‐TaMS interaction by SLC assays and regulation of TaMS expression by BYDV infection. Figure S3: Potential effect of R49S substitution in TaMS2B, growth performance of three TaMS CRISPR mutants, and expression levels of six TaMS members. Figure S4: Comparison of WT Fielder and two derivative HvMS1 OE lines grown under normal conditions. Figure S5: Decreased host defence against TRV caused by mutating NbMS gene members in N. benthamiana. Figure S6: Increased host defence against TRV conferred by overexpressing HvMS1 in N. benthamiana. Figure S7: Interaction of a representative plant MS protein (HvMS1) with the VSRs of five different RNA and DNA viruses. Figure S8: Interaction of HvMS1 with the VSRs of four additional RNA and DNA viruses. Figure S9: Impairment of the anti‐gene silencing function of four different VSRs by HvMS1. Figure S10: Analysis of the structure elements of HvMS1 involved in interacting with VSRs in Y2H assays. Figure S11: Modelling analysis of the interaction between HvMS1 and VSRs. [file PBI-24-3821-s005.docx]

**Support information**

**Methionine synthase positively regulates plant defense to both RNA and DNA viruses and is useful for developing broad-spectrum antiviral resistance in crops**

Zhaohui Wang^1,2,^^†^, Kunpu Zhang^1,2,3,^^†^, Chi Zhang^1,2,†^, Jin Yang^1,2^, Bei Li^1,2^, Lina Wang^1,2^, Zhenghao Shi^1,2^, Rui Guo^1,2^, Shuai Zhang^1,2^, Kaiqi Gao^1,2^, Jianing Li^1,2^, Xiaohuan Jin^1,2^, Xiang Ji^1,2^, Huihui Bi^1,2^, Liyuan You^1,2,*^, Huaibing Jin^4,*^, and Daowen Wang^1,2,^^3,*^

^1^State Key Laboratory of High-Efficiency Production of Wheat-Maize Double Cropping, Henan Center for Crop Genomics and Rice Engineering, College of Agronomy, Henan Agricultural University, Zhengzhou, China

^2^National Wheat Engineering Research Center, College of Agronomy, Henan Agricultural University, Zhengzhou, China

^3^The Shennong Laboratory, Zhengzhou, China

^4^State Key Laboratory for Biology of Plant Diseases and Insect Pests, Institute of Plant Protection, Chinese Academy of Agricultural Sciences, Beijing, China

^†^These authors contributed equally to this study.

**^*^Correspondence authors:** Liyuan You (Tel +86-18697391231; email lyyou@henau.edu.cn), Huaibing Jin (Tel +86-15611108600; email jinhuaibing@caas.cn), and Daowen Wang (Tel +86-18860358017; email dwwang@henau.edu. cn).


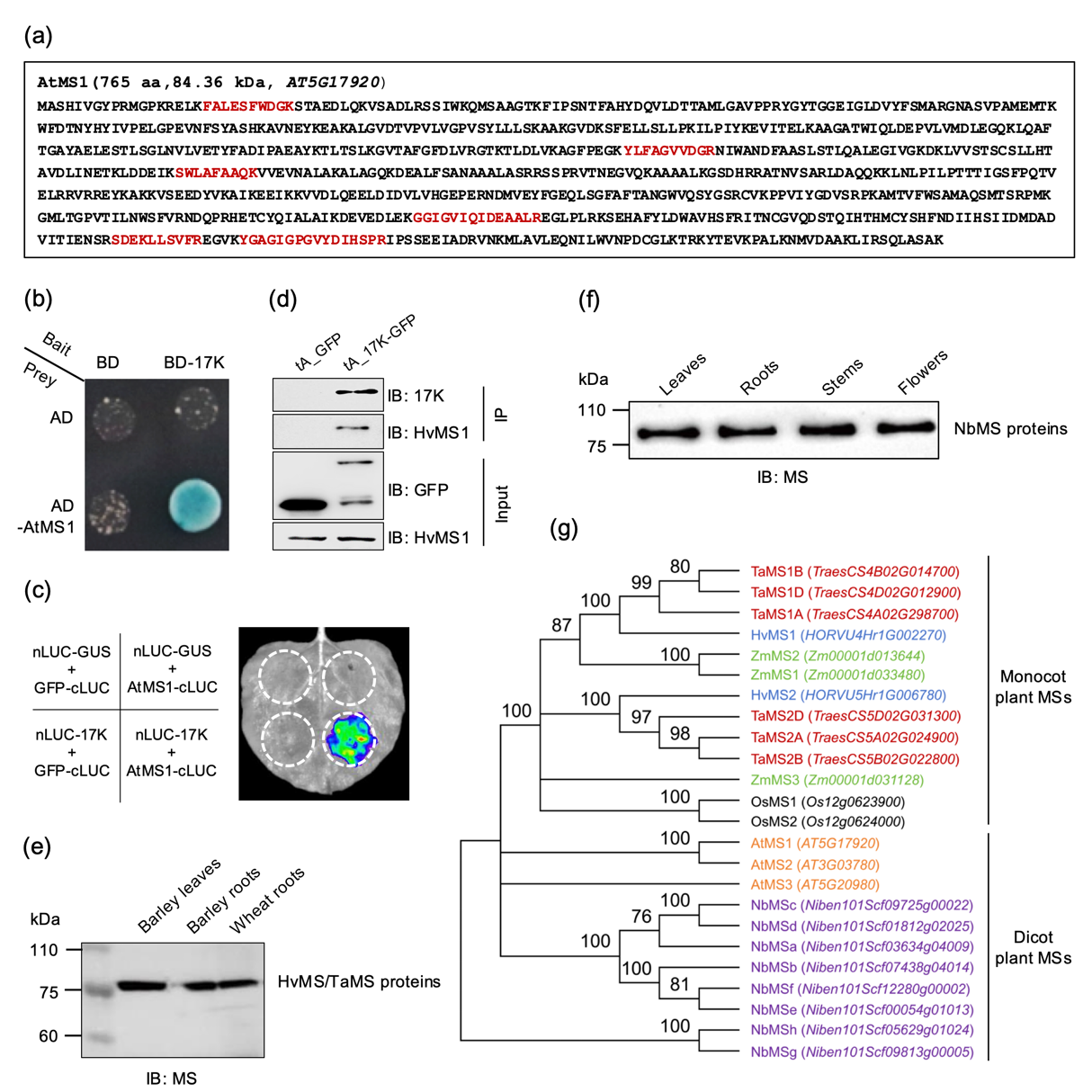


**Figure S1** Identification of AtMS1 as an interacting protein of BYDV 17K VSR. (a) The six unique peptides written in red, identified in our previous IP-MS experiment (Wang et al., 2024), were mapped to AtMS1, which is composed of 765 residues, has a predicted molecular mass of 84.36 kDa, and is encoded by the *Arabidopsis* locus *AT5G17920*. (b-d) Verification of the interaction between BYDV 17K and AtMS1 by Y2H (b), SLC (c), and Co-IP (d) assays. The negative controls were empty AD and BD vectors for the Y2H assays carried out in *Saccharomyces cerevisiae* (b) or nLUC-GUS and GFP-cLUC for the SLC assays conducted in *N. benthamiana* (c). In (d), the immunoprecipitates (IP) were prepared using total proteins extracted from the transgenic lines tA_GFP (expressing free GFP) or tA_17K-GFP (producing 17K-GFP fusion) using the polyclonal anti-MS antibody developed in this study (see below). BYDV 17K was detected in the IP products derived from tA_17K-GFP but not those from tA_GFP. (e, f) A polyclonal antibody, prepared using the bacterially expressed and subsequently purified recombinant HvMS1 protein, could recognize the HvMS/TaMS protein in barley and common wheat tissues (e) as well as the NbMS protein in different *N. benthamiana* tissues (f) in immunoblotting assays. The molecular mass of the TaMS, HvMS, and NbMS proteins detected by the antibody is in the range of their calculated molecular weights (mainly around 84 kDa) (Table S1). (g) Phylogenetic clustering of higher plant MS proteins. The consensus tree shown was developed using neighbor-joining method with 1000 bootstrap permutations. The data presented were representative of three independent experiments.


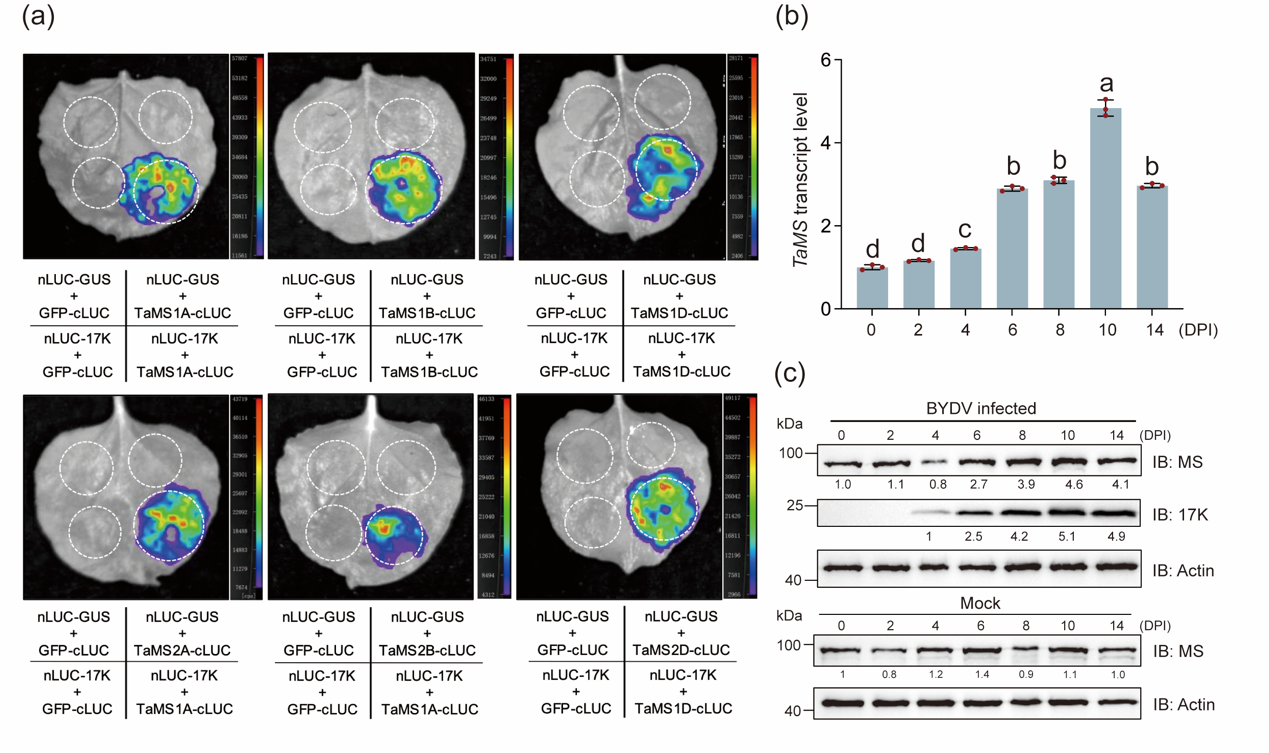


**Figure S2** Detection of BYDV 17K-TaMS interaction by SLC assays and regulation of *TaMS* expression by BYDV infection. (a) The six common wheat TaMS members, TaMS1A, TaMS1B, TaMS1D, TaMS2A, TaMS2B, and TaMS2D, all interacted with BYDV 17K in the SLC assays conducted in *N. benthamiana*, with nLUC-GUS and GFP-cLUC as negative controls. (b, c) Changes of *TaMS* transcript level (b) and TaMS protein abundance (c) at six different time points after BYDV inoculation measured by RT-qPCR and immunoblotting assays, respectively. In (b), the primers used in the assays recognized all six *TaMS* members; the numerical values were each the mean ± SD of three biological replicates; the means were statistically compared using LSD pairwise multiple comparison tests, with different letters indicating significant differences (*P* < 0.05). In (c), the polyclonal anti-MS antibody could detect all six TaMS members (as their protein sequence identities are above 92%, Table S1); protein band intensities were quantified using ImageJ (https://imagej.net/software/imagej/). The results displayed were typical of three independent experiments.


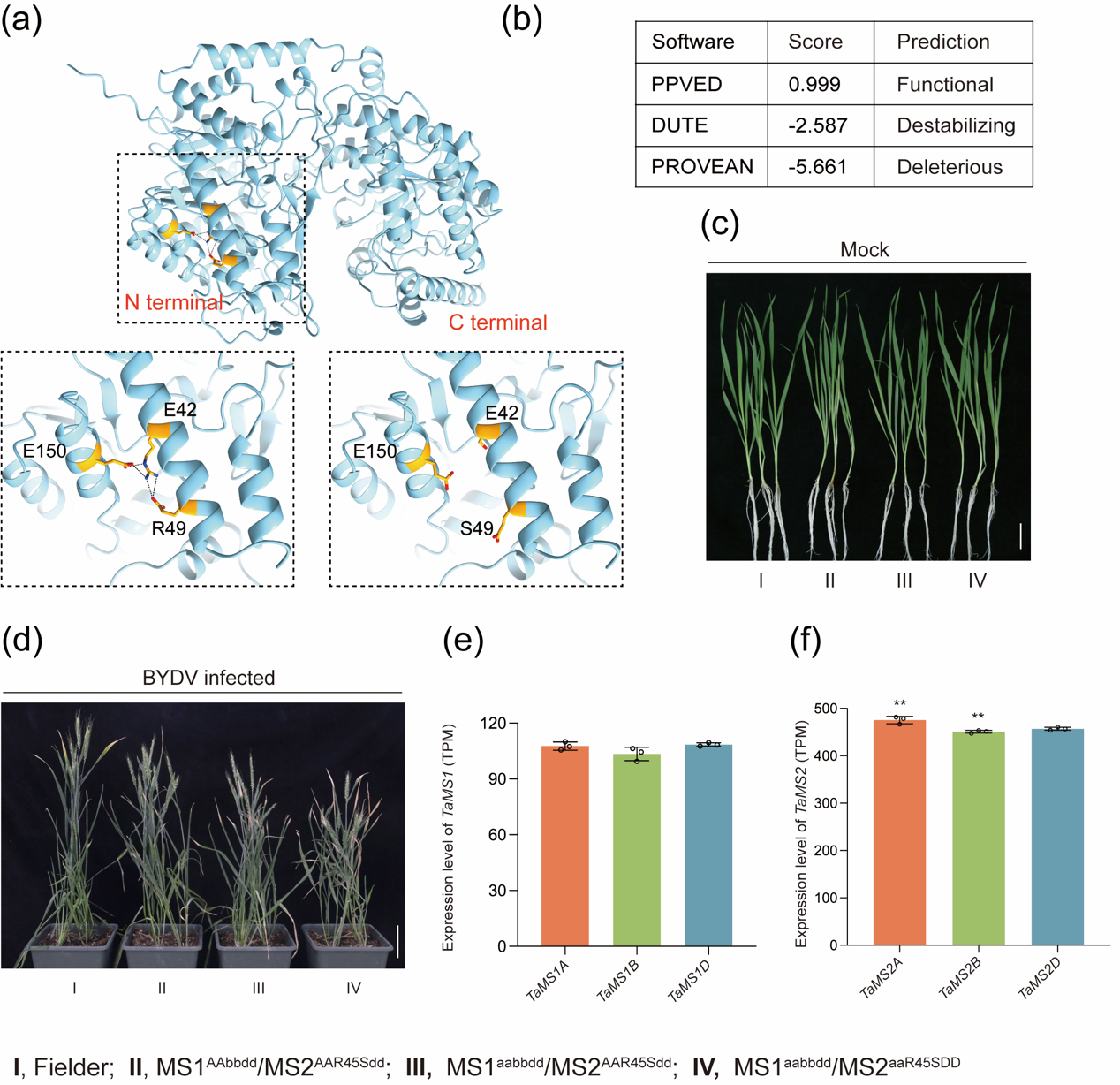


**Figure S3** Potential effect of R49S substitution in TaMS2B, growth performance of three *TaMS* CRISPR mutants, and expression levels of six *TaMS* members. (a) Predicting the effect of R49S substitution in TaMS2B by structural modeling. In the TaMS2B structure predicted by Alphafold3 (https://alphafoldserver.com/), the positively charged Arg49 (R49) forms electrostatic interactions with the negatively charged Glu42 (E42) and Glu150 (E150), thereby stabilizing the N-terminal helix. The R49S mutation disrupts these interactions, likely leading to destabilization of the N-terminal structure and subsequent impairment of protein function. (b) The R49S substitution was predicted to have a functional effect on the protein by PPVED (http://www.ppved.org.cn/), which was likely functionally disruptive based on predictions using the PROVEAN (http://provean.jcvi.org/index.php) and DUTE (https://biosig.lab.uq.edu.au/ duet/) programs. (c) Comparison of plant growth and morphology among WT Fielder and the three genome editing mutants (MS1^AAbbdd^/MS2^AAR49Sdd^, MS1^aabbdd^/MS2^AAR49Sdd^, and MS1^aabbdd^/MS2^aaR49SDD^) of *TaMS* gene members. The plants were grown hydroponically under normal greenhouse conditions, with the photograph taken at five-leaf stage. (d) Comparison of BYDV symptoms in WT Fielder and three genome editing mutants at grain-filling stage. The four genotypes were similarly inoculated with BYDV and allowed to grow to maturity in the greenhouse, with the photograph shown taken at 15 days after flowering. (e, f) The expression levels of *TaMS1* (e) and *TaMS2* (f) gene members in common wheat shoot tissues. This analysis used the RNA sequencing data (SRP028357) deposited in the Wheat Expression Browser (http://www.wheat-expression.com/), which were generated using three biological replicates of the shoot tissues of the common wheat variety Chinese Spring (SRR946458, SRR946459, and SRR946460). Scale bar, 3 cm (c) or 10 cm (d). The numerical values shown were each the mean ± SD of three independent replicates. Statistical analysis was performed using the Student’s *t*-test. **, *P* < 0.01.


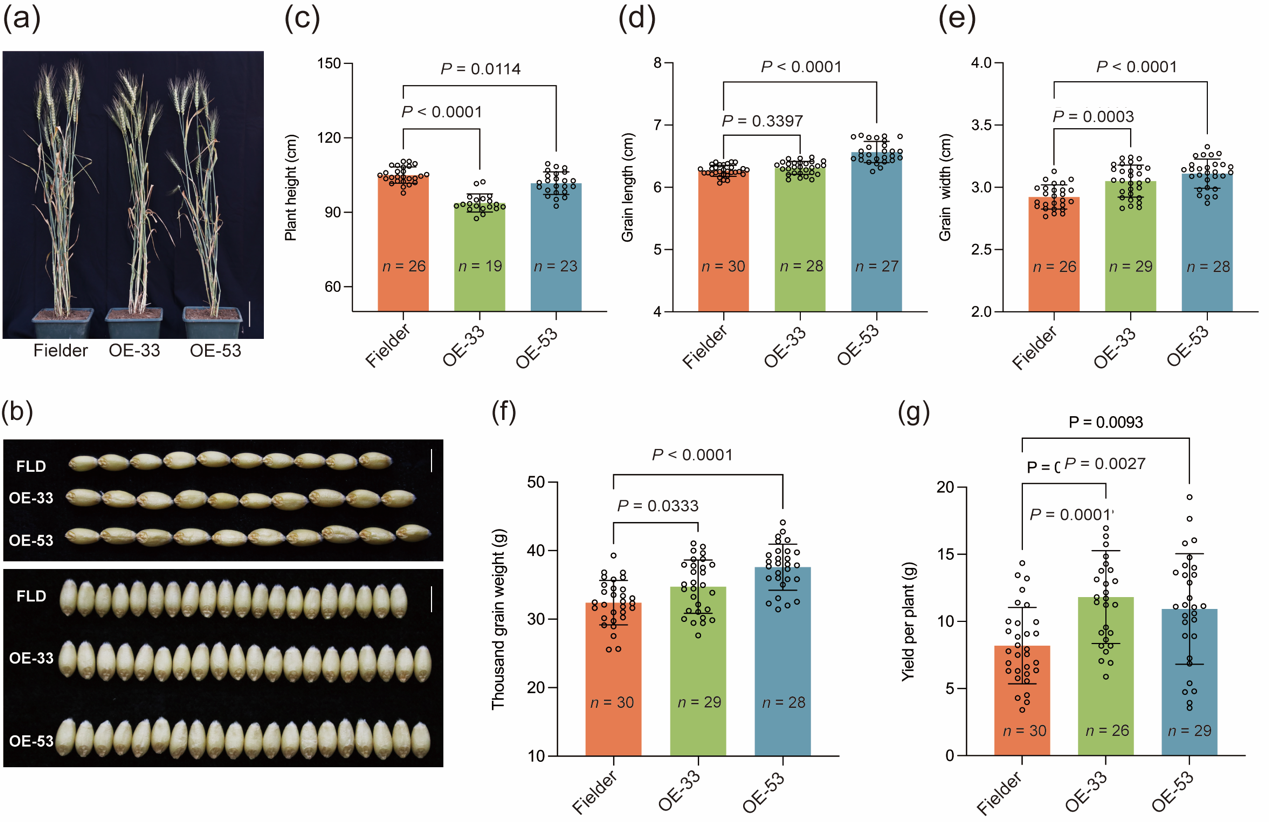


**Figure S4** Comparison of WT Fielder and two derivative HvMS1 OE lines grown under normal conditions. (a, b) Evaluation of plants (a) and grains (b) among the three genotypes. Scale bar, 10 cm (a) or 0.5 cm (b). (c-g) Quantitative evaluation of plant height, grain length, grain width, thousand grain weight, and yield per plant among the three genotypes. The numerical values shown were each the mean ± SD of 19 plants. Statistical analysis was performed using the Student’s *t*-test.


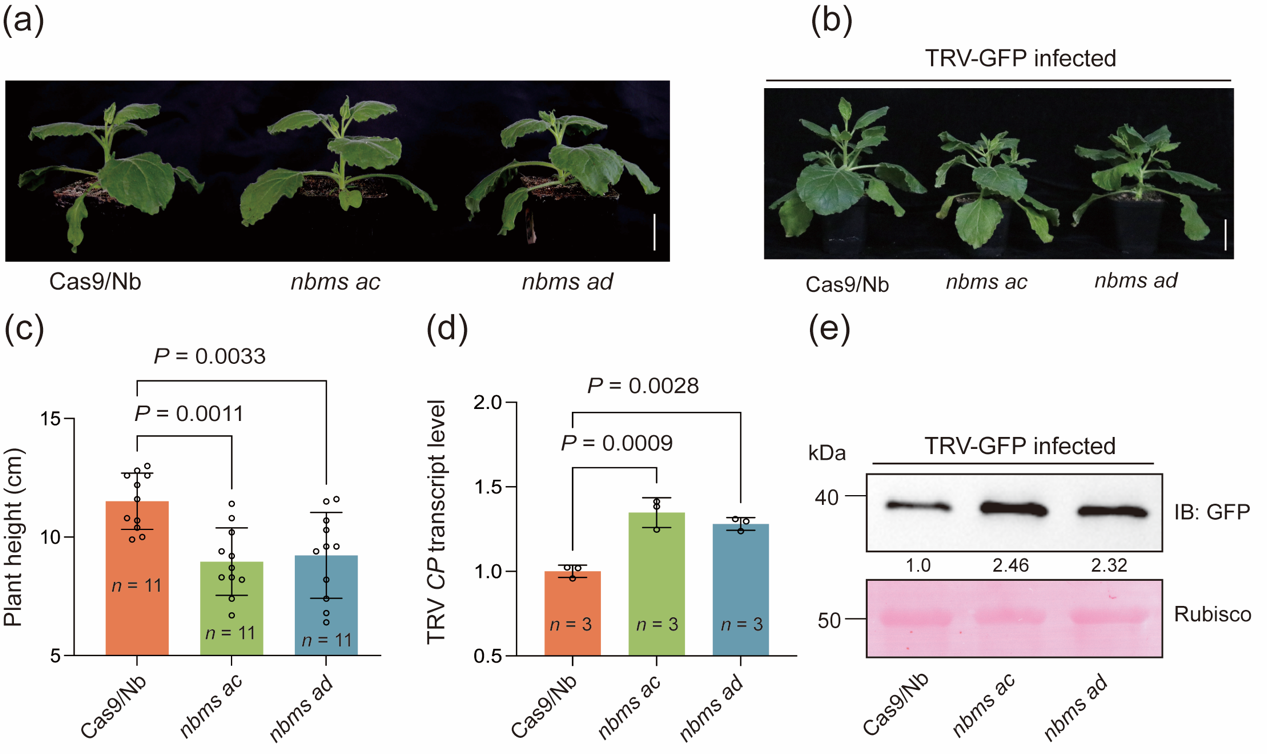


**Figure S5** Decreased host defense against TRV caused by mutating *NbMS* gene members in *N. benthamiana*. (a) Similarity among the control (Cas9/Nb) and two derivative *NbMS* genome editing mutants (*nbms ac* and *nbms ad*) cultured under normal greenhouse conditions. (b, c) Morphological differences (b) and changes of plant height (c) among the control and two *nbms* double mutants examined at 14 DPI of TRV-GFP. (d, e) The three types of plants shown in (b) were analyzed for the transcript level of TRV coat protein gene (*CP*) (d) and TRV mediated GFP expression (e) at 14 DPI by RT-qPCR or immunoblotting assays. Scale bar, 5 cm. The values were means ± SD of at least 3 plants (c) or three biological replicates (d) for each genotype, with the means compared using Student’s *t*-test. In (e), ponceau S staining of Rubisco large subunit served as a loading control; GFP band intensities were quantified by ImageJ. The results shown were representative of three independent experiments.


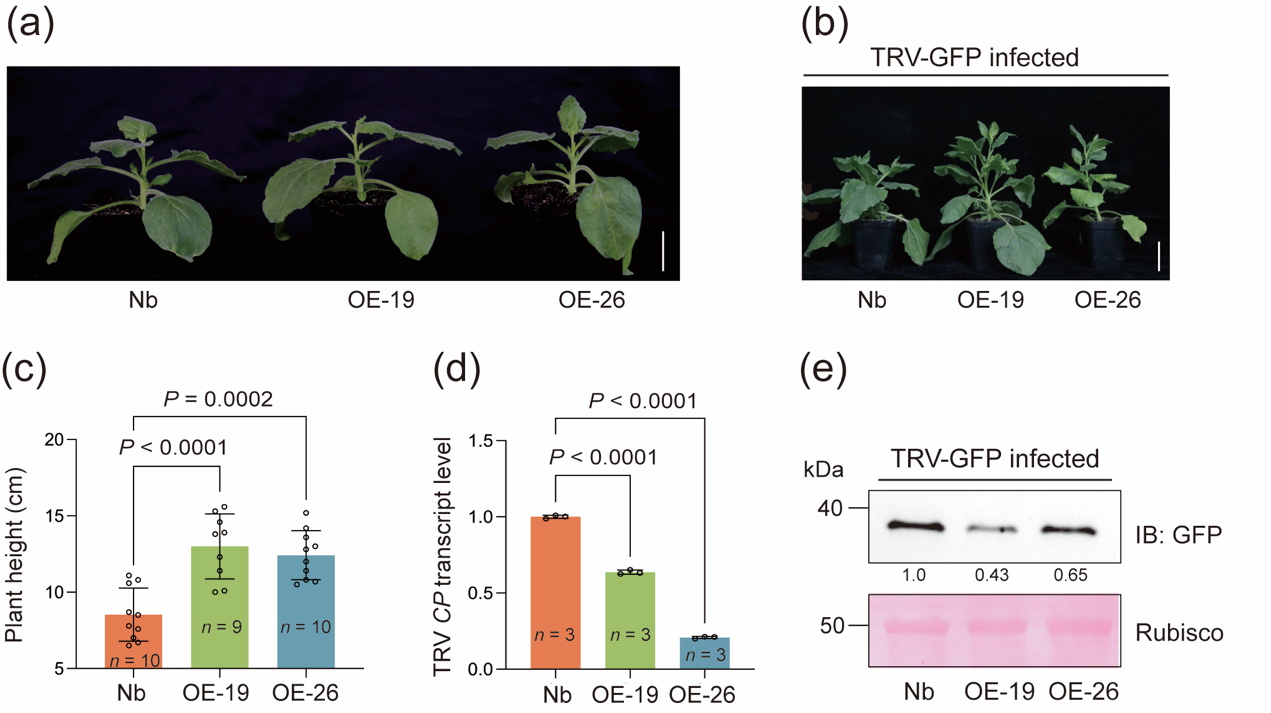


**Figure S6** Increased host defense against TRV conferred by overexpressing *HvMS1* in *N. benthamiana*. (a) Similarity among the control (Nb) and two derivative *HvMS1* overexpressing lines (OE-19 and OE-26) reared under normal greenhouse conditions. (b, c) Morphological differences (b) and changes of plant height (c) among WT control and the two different OE lines expressing HA-tagged HvMS1 protein analyzed at 21 DPI. (d, e) The three types of plants shown in (b) were examined for the transcript level of TRV coat protein gene (*CP*) (d) and TRV mediated GFP expression (e) at 21 DPI by RT-qPCR or immunoblotting assays. Scale bar, 5 cm. The values were means ± SD of at least 3 plants (c) or three biological replicates (d) for each genotype, with the means statistically compared using Student’s *t*-test. In (e), ponceau S staining of Rubisco large subunit served as a loading control; GFP band intensities were quantified by ImageJ. The results shown were reproducible in three independent experiments.


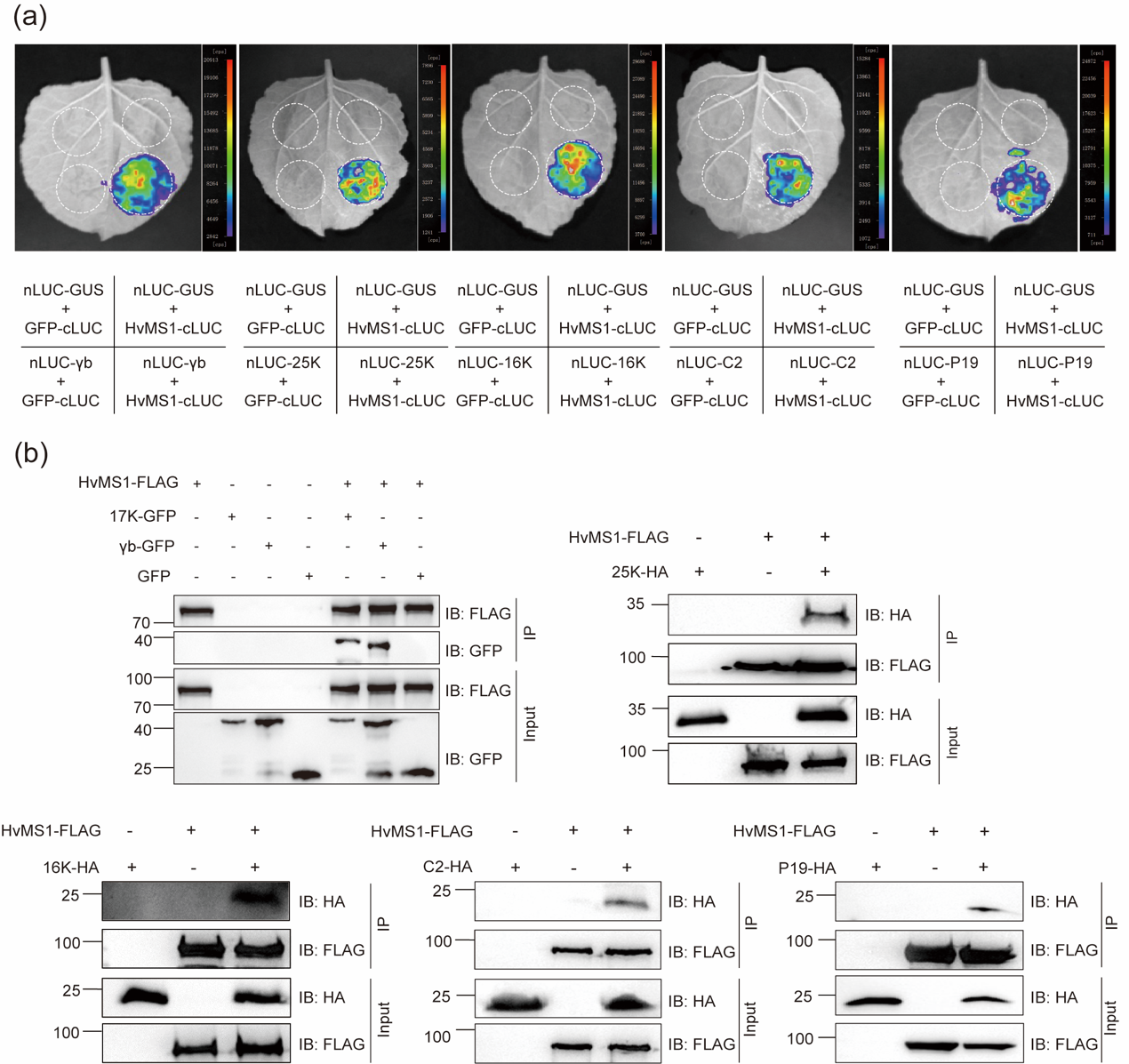


**Figure S7** Interaction of a representative plant MS protein (HvMS1) with the VSRs of five different RNA and DNA viruses. (a) SLC assays showing the interaction of five VSRs (BSMV γb, PVX 25K, TRV 16K, BSCTV C2, and TBSV P19) (Figure 7a and Table S2) with HvMS1. The negative controls were nLUC-GUS and GFP-cLUC in these assays. (b) Verification of the interaction between HvMS1 and five VSRs by Co-IP assays. *Agrobacterium* strains carrying the constructs expressing FLAG-tagged HvMS1 or HA-tagged VSR (γb, 25K, 16K, C2, or P19) were co-infiltrated into the foliar cells of *N. benthamiana*. Total proteins, extracted from the infiltrated tissues at 48 h later, were subject to immunoprecipitation (IP) with anti-FLAG antibody. Presence of VSR in the IP products was investigated by immunoblotting with anti-HA antibody. The results displayed were representative of three independent experiments.


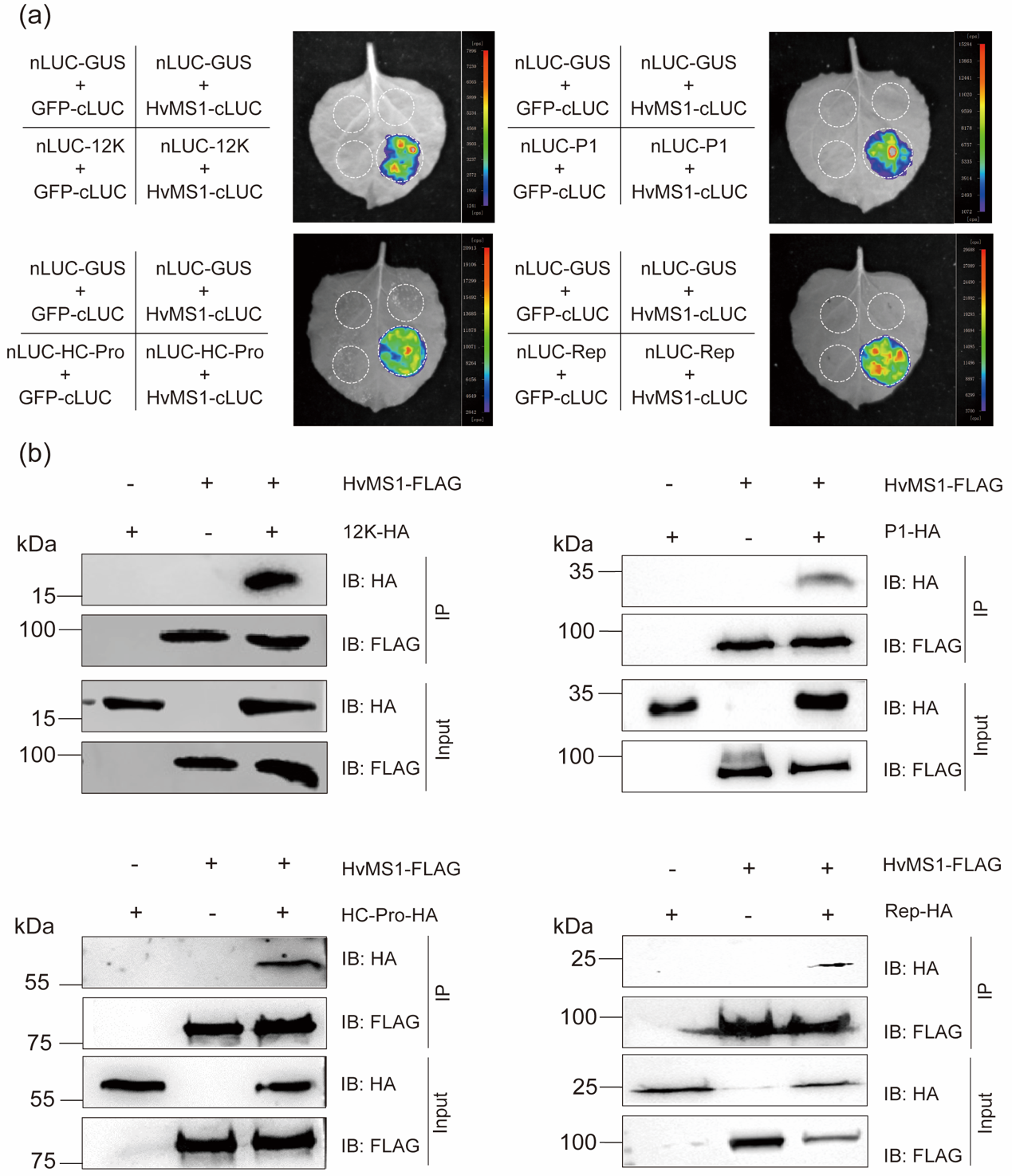


**Figure S8** Interaction of HvMS1 with the VSRs of four additional RNA and DNA viruses. (a) Interaction of four VSRs, namely PEBV 12K, WYMV P1, TuMV HC-Pro, and WDV Rep (Figure 7a and Table S2), with HvMS1 revealed by SLC assays. The nLUC-GUS and GFP-cLUC were used as negative controls in these assays. (b) Validation of the interaction between HvMS1 and the four VSRs by Co-IP assays. *Agrobacterium* strains carrying the constructs expressing FLAG-tagged HvMS1 or HA-tagged VSR (12K, P1, HC-Pro, or Rep) were co-infiltrated into *N. benthamiana* leaf cells. Total proteins, prepared from the infiltrated tissues at 48 h later, were subject to immunoprecipitation (IP) with anti-FLAG antibody. Existence of VSR in the IP products was detected by immunoblotting with anti-HA antibody. The results depicted were representative of three independent experiments.


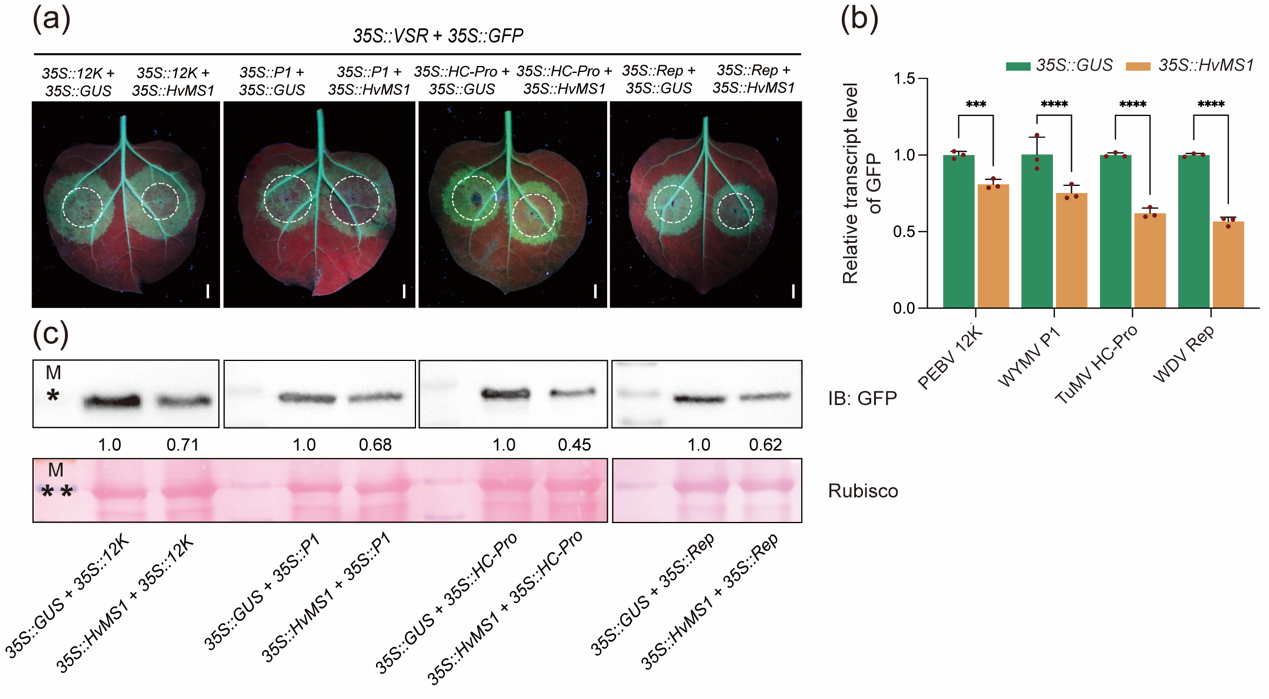


**Figure S9** Impairment of the anti-gene silencing function of four different VSRs by HvMS1. (a) Inhibition of the anti-gene silencing function of four VSRs (PEBV 12K, WYMV P1, TuMV HC-Pro, and WDV Rep) by HvMS1, which were carried out in the 16c *N. benthamiana* line. *Agrobacterium* strains carrying the *35S::GUS* or *35S::HvMS1* construct were first infiltrated into the left and right halves of the leaves of 16c tobacco, respectively. At 24 h later, *Agrobacterium* strains carrying the *35S::GFP* or *35S::VSR* construct were co-infiltrated into the same sites, with the infiltrated areas being larger than those formed in the initial agroinfiltration (marked by dashed circles). GFP fluorescence was recorded at three days post the second infiltration. Transient expression of HvMS1 (in the right circled area), but not that of GUS (in the left circled area), weakened the anti-gene silencing function of the four VSRs. (b, c) Effects of infiltrating *35S::GUS* or *35S::HvMS1* construct on the *GFP* transcript level (b) and GFP protein abundance (c) in the presence of different VSRs. The RT-qPCR or immunoblotting assays were conducted at three days post the second infiltration. In (b), the relative values, normalized to that obtained for *35S::GUS* (set as 1), were means ± SD of three biological replicates. In (c), GFP band intensities were quantified using ImageJ. The molecular mass standards labelled by * and ** in the marker lane (M) were 30 and 55 kDa, respectively. Statistical analysis was performed using Student’s *t*-test (****, *P* < 0.001). The results shown were representative of three independent experiments.


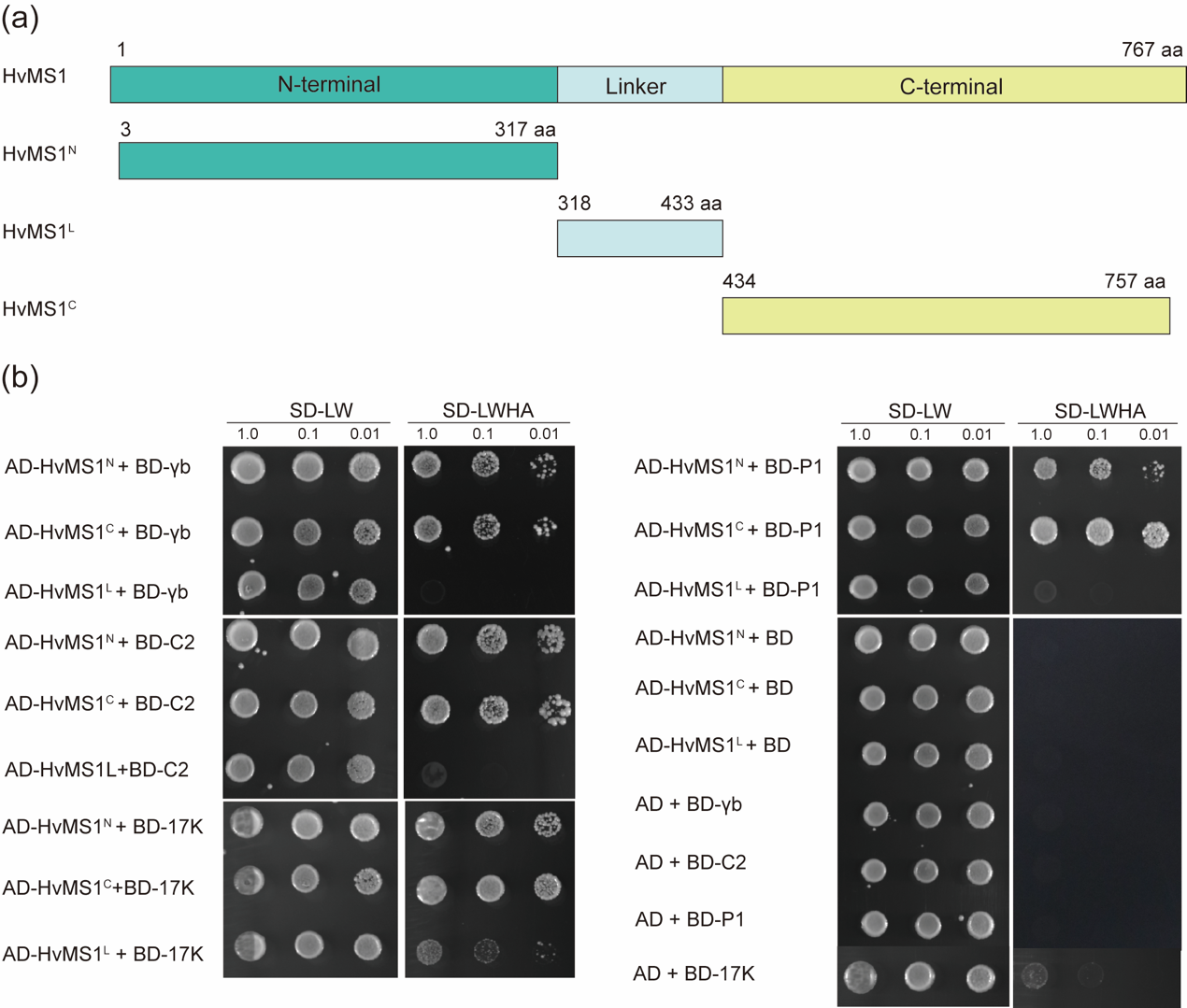


**Figure S10** Investigation of HvMS1 structural elements involved in interacting with VSRs using Y2H assays. (a) Schematic representation of full-length HvMS1 protein (767 aa) and three derivative deletion mutants, i.e., HvMS1^N^ (amino acids 3 - 317aa, the N-terminal domain), HvMS1^L^ (amino acids 318 - 433aa, the linker region), and HvMS1^C^ (amino acids 434 - 757aa, the C-terminal region). (b) Y2H assay results obtained using three HvMS1 deletion mutants as preys and four presentative VSRs (γb, C2,17K, and P1) as baits. The data shown were representative of three independent experiments.


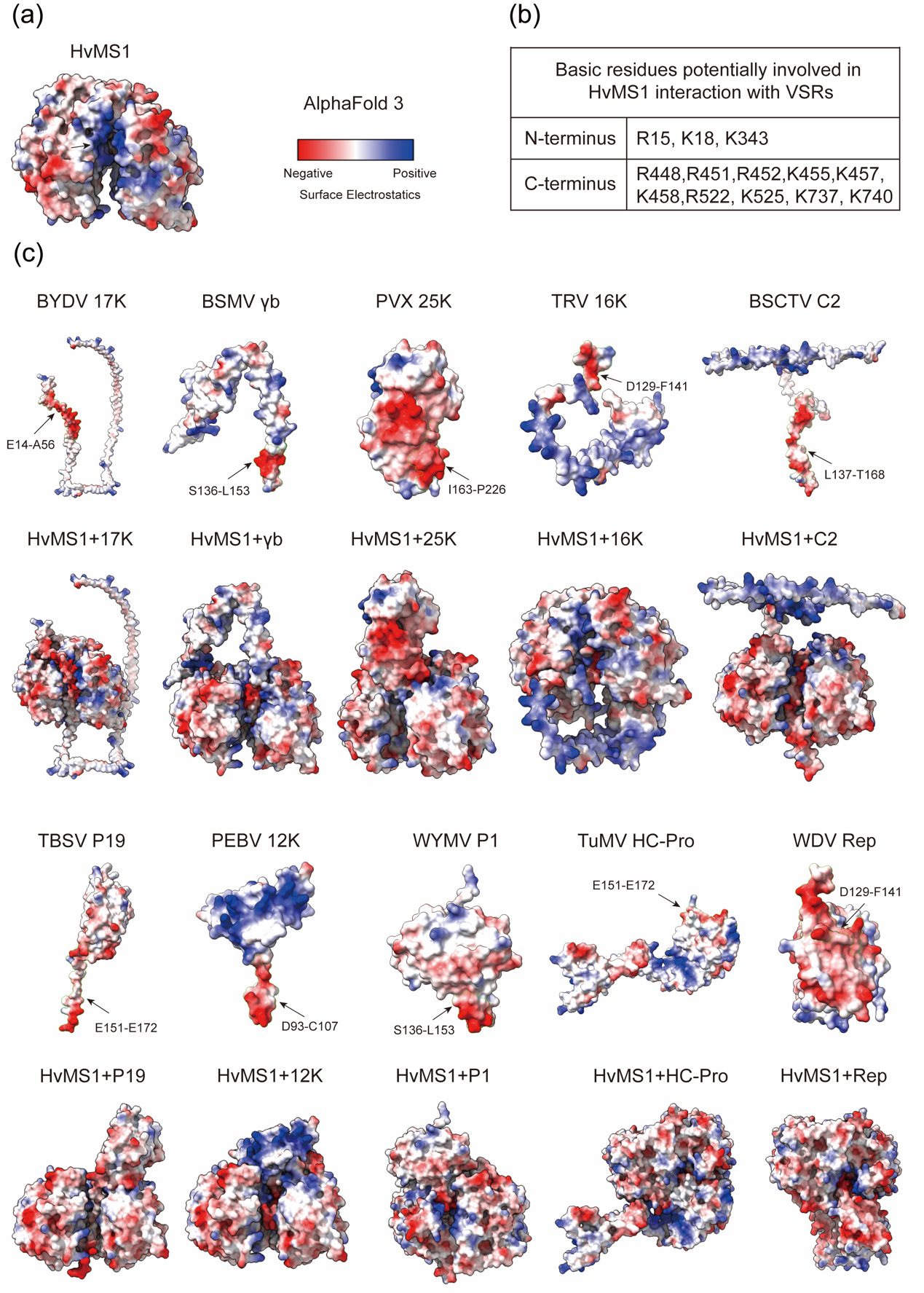


**Figure S11** Modeling analysis of the interaction between HvMS1 and VSRs**.** (a) Putative electrostatic potential map of the HvMS1 structure predicted with AlphaFold 3. A positive electrostatic patch (arrowed) exists in the inter-barrel region of the predicted HvMS1 structure. (b) Basic residues (Arginine and lysine) involved in the formation of the positive electrostatic patch shown in (a). (c) Presence of negative electrostatic patch (arrowed) in each of the 10 VSRs and predicted interaction complex between HvMS1 and each VSR. Electrostatics were modelled using the software ChimeraX.
